# A Compact Methodology to Understand, Evaluate, and Predict the Performance of Automatic Target Recognition

**DOI:** 10.3390/s140711308

**Published:** 2014-06-25

**Authors:** Yanpeng Li, Xiang Li, Hongqiang Wang, Yiping Chen, Zhaowen Zhuang, Yongqiang Cheng, Bin Deng, Liandong Wang, Yonghu Zeng, Lei Gao

**Affiliations:** 1 School of Electrical Science and Engineering, National University of Defense Technology, 137 Yanwachi Street, Changsha 410073, China; E-Mails: lixiang01@vip.sina.com (X.L.); oliverwhq@tom.com (H.W.); ypchenhk@gmail.com (Y.C.); zwzhuang@nudt.edu.cn (Z.Z.); nudtyqcheng@gmail.com (Y.C.); dengbin@nudt.edu.cn (B.D.); 2 State Key Laboratory of Complex Electromagnetic Environment Effects on Electronics and Information System (CEMEE), Luoyang 471003, China; E-Mails: CEMEE@vip.163.com (L.W.); lotus2seeds@gmail.com (Y.Z.); ren_lgao@126.com (L.G.)

**Keywords:** automatic target recognition, performance evaluation, performance prediction

## Abstract

This paper offers a compacted mechanism to carry out the performance evaluation work for an automatic target recognition (ATR) system: (a) a standard description of the ATR system's output is suggested, a quantity to indicate the operating condition is presented based on the principle of feature extraction in pattern recognition, and a series of indexes to assess the output in different aspects are developed with the application of statistics; (b) performance of the ATR system is interpreted by a quality factor based on knowledge of engineering mathematics; (c) through a novel utility called “context-probability” estimation proposed based on probability, performance prediction for an ATR system is realized. The simulation result shows that the performance of an ATR system can be accounted for and forecasted by the above-mentioned measures. Compared to existing technologies, the novel method can offer more objective performance conclusions for an ATR system. These conclusions may be helpful in knowing the practical capability of the tested ATR system. At the same time, the generalization performance of the proposed method is good.

## 1. Introduction

### 1.1. The ATR Technology and Performance Analysis for It

Automatic target recognition (ATR) is the capability for an algorithm or equipment to recognize targets or objects based on the data obtained from sensors [1,2]. ATR is an essential component of intelligence systems implemented with various types of sensors [1,3]. Therefore, it is of great importance to have an objective and quantitative performance evaluation measure for an ATR system [1].

ATR technology is widely employed as the essential technique in advanced systems such as within the military [4,5], security [6,7] and modern medical science [8]. It enables a radar to catch its object of interest [9,10], helps a seeker find the fixed target in a complicated scenario [3,11], and makes the accurate diagnosis possible with medical sensors [12,13]. Nowadays, ATR is frequently typified by the application of radars and optical-sensors [4].

The primary principle in ATR is inverse theory, which, enlightened by the feeding signal collected with certain types of sensors, makes decisions on the information related to the intended target [3,9] (see “ATR system” in figure of Section 2.1). For example, people may know that ATR can be viewed as an inverse problem in the fields of electromagnets and acoustics: targets of interest are sensed, the sensed signatures are then transmitted to the detectors, and the main purpose of ATR is to use these signatures to classify the original targets [1,5,14].

As for the components of an ATR system, the employed sensor can be a polarimetric infrared, a hyperspectral device, or an ultra-wide band radar [3]. Many kinds of classifiers are investigated, such as model-based classifier, statistical based classifier, phenomenological modeling classifier, context information based classifier and information fusion classifier [9]. With the rapid advances in sensor technology, flexible field programmable gate array (FPGA), high performance computer, the art of ATR is becoming more pertinent to a much wider group of scientist and engineers than before [1,14]. As we proceed into the future, there will be more and more research/applications of ATR technologies and ATR systems [2].

However, given the changing environment and the limitation of the sensors, this system sometimes runs into trouble [15]. For example, the same kind of cell and the diseased tissue being observed may vary in shape, size and even quality [16,17], the vehicles being investigated may shift in velocity, pitch and direction [6], a certain type of sensor can only collect limited information from the target [3], and this point is further complicated by the fact that so many systems and factors are involved in the signal processing course of an ATR system [11].

Given the facts mentioned previously, the performance evaluation for ATR systems (PE-ATR) and the performance prediction for ATR systems (PP-ATR) continue to be studied by many experts in the field [18,19]. As aforementioned, the application of ATR in radars and photo-sensors is frequently found. Consequently, the literature on performance evaluation in those ATR systems maintains the major part in this area [20,21]. There are evaluation technologies for ATR in radar systems [22,23], performance assessment work on ATR algorithms employing motion imagery [24], performance prediction testbed for an image-based ATR algorithm [20], *etc.* When reviewing the available technologies, most of the literatures are from the radar and photo-sensor related discipline. Typical performance evaluation and performance prediction technologies for an ATR system are as follows.

### 1.2. Scaling the Operating Condition for the ATR System

ATR systems operate and are tested under certain conditions. These conditions may be regarded as subsets of a multi-dimensional space of conditions [25]. To obtain an objective conclusion, the operating condition should be considered in the performance evaluation course [9]. However, the scenarios of the ATR system are many and varied [26]. It is of great importance to develop some approaches to scale the operating condition (throughout this paper, unless otherwise stated, “operating condition” is all the scenarios an ATR system can be applied) for an ATR system [27]. Unfortunately, the literature on scaling the operating condition for an ATR system is limited. Available works are as follows:

- Operating condition description with concepts. Generally, there are four sets of conditions: operating (here, “operating condition” is only the operational condition), testing, modeled, and training [25]. The relation of them can be shown in a Venn diagram [25]. Although this approach is not a quantified way, it is helpful in discussing the performance of an ATR system.
- For image metric-based ATR and synthetic aperture radar (SAR) ATR, the operating condition is sometimes quantified in the way of image characterization [28]. As a fundamental idea, the concept of “Extended Operating Condition (EOC)” is defined [27,29]. EOC is an operating condition “away” from the trained condition [27]. Experiments shown the tested SAR ATR performance (recognition rate) was very sensitive to the EOCs tested [27,30].
- With respect to the condition in signal processing in ATR, amplitude affection factor (AAF) and signal to noise ratio affect factor (SNRF) are developed [9]. In view of the condition of feature extraction, extend of recognition (ER) is proposed [9]. These metrics are further applied in denning performance evaluation indexes and building performance evaluation models [9].

### 1.3. Performance Evaluation for ATR Systems

As mentioned above, the performance evaluation for ATR systems is studied by many experts. The available technologies can be divided into two main groups according to the framework: model-based and data-based approaches [9]. When estimating the performance for an ATR system, model-based approaches usually work with a performance model such as the expected measures of effectiveness (MOE), robust evaluation model and independence evaluation model [9,19]. The data-based approaches directly calculate some indexes from the recognition output such as recognition rate and false alarm [21]. In practice, these two approaches are often combined in performance evaluating for an ATR system.

#### 1.3.1. Basic Performance Evaluation Indexes

In PE-ATR, some basic measures like probability of detection (*P*_D_), recognition rate (RR), and false alarm rate (*P*_FA_) are generally employed facilities [9]. Estimating the performance bound is concerned in the early years [31].

Performance concepts are also introduced to compare performance across ATR technologies [25]. Two classes of concepts are proposed [25]. One class is referred to as performance. It includes accuracy, extensibility, robustness, and utility [25]. These Performance concepts consider the relationship between the test data, the training data, and data from modeled conditions [25]. The other class is called cost. It includes efficiency, scalability, and synthetic trainability [25]. The latter class of concepts put the cost into three categories: data-collection, data storage, and data-processing [25].

The confusion matrix (CM) is another widely used performance evaluation approach [19,32]. It can be easily configured and employed for a diverse set of ATR systems. The matrix is a square grid with a single row and a single column corresponding to each category defined in the data set. The (*i*, *j*) cell in the matrix is the number of predicted classifications on category *j* that correspond to the truth source of category *i* [19].

It should be noted that, in some related works, the quantities to show the performance of an ATR system are all referred to as “performance metric” or “character of performance”. However, they are referred to as “performance indexes” in this work hereafter.

#### 1.3.2. Performance Evaluation Based on Performance Modeling

In PE-ATR, many scientists work with performance models and/or evaluation models [33]. The existing performance models and evaluation models can be classified as: (a) models based on probability, statistics, and random processes [9,34]; (b) models based on Bayesian approach [35]; (c) models based on information theory approach [35]; (d) subsystem performance models [36]; (e) other performance models [37].

(a) Models Based on Probability, Statistics, and Random Processes
  Series of performance indexes are built based on probability, statistics, and random processes: measurement of recognition rate (MRR), measurement of false recognition rate (MFRR), mean of MRR, variance of MRR, the independence of MRR to operating condition, *etc.* [9].
(b) Models Based on Bayesian Approach
  As for the Bayesian approach, probability distributions are used to represent the variability in target and background signatures [35]. To apply the method, assumptions are usually made (such as the use of Gaussian distributions and independence of information sources) to ensure mathematical tractability. However, these assumptions are not always practical enough [35].
(c) Models Based on Information Theory Approach
  This kind of model casts the recognition problem as a communication process [35]. Information theory brings in the notation of entropy and measures of relative information to try to figure out how information and thus performance is lost along the processing course [35]. It may suffer from the problem when assumptions do not match reality closely enough. This kind of performance indexes have been applied in evaluating SAR ATR [38].
(d) Subsystem Performance Models or Performance Model for Certain Metric
  The computational burden is an important metric for image recognition [16]. It is further considered for image recognition of high resolution radar sensors [16].
  For SAR ATR, polarization and resolution may affect the performance [36,39]. This can be studied with the help performance curves (probability of detection to false alarms) [36]. For some ATR algorithms, performance curves at all three ATR stages (detection, discrimination, and classification) for certain combination of polarization and resolution were studied by the Lincoln Laboratory [36,39].
  Performance evaluation of the subsystem of an ATR system is meaningful. The reliability analysis of the sensor employed in ATR is of interest [40]. Performance indexes are built on two fundamental issues: reasonable dissimilarity among evidences, and adaptive combination of static and dynamic discounting [40]. These measures are helpful to optimize the mentioned ATR algorithm [40].
(e) Other Performance Models
  To study the potentialities of polarimetric SAR interferometry (POLInSAR) in developing a new classification methods for ships, performance evaluation has been performed to accomplish a trade-off between geometry description accuracy and method robustness in reference feature vectors (or patterns) [37]. Experiments showed a low number of vectors could lead to an overestimation of the classification rate, and an excessive number of patterns would make quite similar geometries to be classified in different classes [37].

#### 1.3.3. Receiver Operating Characteristic Analysis and Similar Approaches

Receiver operating characteristic (ROC) analysis is a broadly used performance analysis tool in signal processing and communications [34,41]. Researchers have introduced this notation into PE-ATR. A three-dimensional (3-D) ROC trajectory was presented to compare competing target recognition algorithms when unknown targets are present in the data [34]. In understanding the tradeoffs between the probability of rejection and other two performance measures commonly used in detection problems, it is a useful tool for SAR image analysis [34,42].

Scientists also extended the conventional ROC analysis from single-signal detection to detection and classification of multiple signals [41,43]. Applications showed it was a flexible utility in PE-ATR [41].

An extension of the ROC method is the analysis of performance bounds in different scenarios [15]. Some analytical characters on PE-ATR are obtained under complicated, non-Gaussian models and optimized system parameters [15]. For targets composed of a constellation of geometrically-simple reflectors, lower and upper bounds on the probability of correct classification are estimated in SAR ATR [44,45]. In performance evaluation for sidescan sonar target classification, some common bounds are derived to show the properties of ATR [46]. In pose estimation related to ATR, Hilbert-Schmidt lower bounds for estimators on matrix Lie groups is defined and validated [47].

Another extension of ROC method is confidence intervals for ATR performance evaluation index [48,49]. The provided confidence interval estimator includes proportion estimation based on Binomial distribution and rate estimation based on Poisson distribution. Under the Bayesian posterior distribution, this estimator is substantially more accurate than other similar approaches [48].

Automatic fingerprint recognition is a interdisciplinary field. It includes image processing, pattern recognition, computer technology, and so on. The confidence interval is compared between different automatic fingerprint recognition algorithms [50]. A performance model is built based on statistics. It can be applied to estimate the uniqueness of the template in classifiers [50].

#### 1.3.4. Performance Evaluation Framework

Performance evaluation indexes assess the capability in various aspects. However, people sometimes seek an integrated conclusion in some different sides [21]. Therefore, performance evaluation framework is concerned and investigated. Generalized performance model is built based on fuzzy comprehensive evaluation, fuzzy integration and fuzzy cluster analysis [9]. These performance models can offer an algorithm-independent view of the ATR performance [21].

#### 1.3.5. Other Performance Evaluation Methods

Underwater target recognition is challenging due to the presence of noise, point-spread function effects resulting from camera or media inhomogeneities [51]. Image compression transform is sometimes applied. Performance evaluation method of data compression transforms is then developed to achieve low-distortion images that eases the burden of classifiers [51].

For automatic face recognition systems, the effect of racial and gender demographics on estimating the accuracy of algorithms is considered [52]. It was reported that differences in the match threshold was required to obtain a false alarm rate of 0.001 when demographic controls on the non-matched identity (race or gender) pairs varied [52].

#### 1.3.6. Performance Evaluation System or Performance Evaluation Testbed

As for PE-ATR software or a testbed, an example is given where Python (an open source scripting language) and OpenEV (a viewing and analysis tool) have been incorporated [53]. This testbed gives important insight into the risks as well as the successful use of open source language in ATR [53].

An experimental system called automated instrumentation and evaluation (Auto-I) is developed [32]. Auto-I has a module for automatic adaptation of algorithms parameters using algorithms performance models, optimization and artificial intelligence techniques [32]. The presented design of Auto-I is modular, it can be interfaced to other ATR systems except for the ATR system in [32].

For image-based target detection, a complete truthing system is developed [54]. It is named “the Scoring, Truthing, And Registration Toolkit (START) [54]”. This toolkit can align the images of the identical scene to a common reference frame. Then, “truthing” is applied to specify target identity, position, orientation, and other scene characteristics [54]. Finally, “scoring” is used to evaluate the performance of certain algorithms as compared to the specified truth [54].

### 1.4. Performance Prediction for ATR Systems

Compared to performance evaluation work, the existing performance prediction methods are fairly limited [20]. To some extend, the available performance prediction methods are extending work of PE-ATR.

#### 1.4.1. Basic Performance Prediction Approaches for ATR Systems

Based on image measures quantifying the intrinsic difficulty of ATR, a performance forecaster is developed [20]. The performance measures include: constant false alarm rate (CFAR), power spectrum signature, probability of edge, etc. This algorithm offers a method for predicting ATR performance based on information extracted directly from the imagery [20]. The statistical accuracy is another basic index in performance predicting [55].

A generally employed performance prediction index is performance bound, namely, upper bound [56] and lower bound [21]. In this approach, the frequently considered performance include: detection rate, false alarm rate and recognition rate.

#### 1.4.2. Performance Prediction Based on Performance Modeling

When predicting the performance for an ATR system, performance models are widely employed [20]. Simple models are easy to configure, but they cannot accurately quantify performance [57]. Detailed models may freely respond to the scenario, however, the detailed models are difficult to investigate [35,57].

When the features are distorted by uncertainty (occlusion and/or clutter) in both feature locations and magnitudes, the performance of an ATR system is especially difficult to predict. A practical way is to estimate the performance bound for the system [57]. For a vote-based object recognition system, forecasting lower and upper bound recognition ability is implemented [57]. This approach takes object model similarity into account, so that when models of objects are more similar to each other, then the desired recognition rate is lower [57].

The parameters of ATR algorithms can be used for predicting the performance for an ATR system [58]. The levels of robustness and invariance of parameters are employed as predictive indicators of ATR performance along with self refusal capabilities of the ATR algorithms [58].

A model of the subsystem of an ATR system can be introduced in forecasting the performance for the system [59]. One of the methods models the capability of the classifier. The classifier is based on a Bayes match between vector of extracted scattering features and a vector of predicted features. Uncertainty in both extracted and predicted features are included in the match metric (evaluation index) [59]. With scattering centers extracted from measured SAR imagery of ten targets, experiments show that the proposed match metric (evaluation index) is helpful in predicting the performance for an ATR system [59].

To estimate and predict the computational error of an ATR system, scientists developed error probability distribution method [60,61]. It is resolved from error function that is derived from the parse tree which represents a given ATR algorithm [60,61]. Field tests of performance prediction were performed in terms of computational accuracy, cost, and portability. The results show the prediction is reasonable [60,61].

Algorithm-independent predicting of the ATR performance is highly welcomed. To facilitate evaluation of performance tradeoffs for SAR designs, performance predictions are performed including both parameter selections (e.g., bandwidth and transmit power) and added domains of SAR observation, such as 3-D, full polarimetry, aspect diversity, and/or frequency diversity [62]. Discussion is made about performance of 3-D SAR includes parameter tradeoffs of various height resolutions at the target, and various numbers of sensors [62]. This work is significant in supporting SAR ATR designation.

#### 1.4.3. Other Performance Prediction Method

To optimize the speech recognition performance in a computer assisted language learning system, a decision tree-based method is incorporated to predict possible speaking errors made by non-native speakers [63]. Trials of the language learning system and the performance prediction were conducted [63]. Positive feedback was reported [63].

The confidence interval is compared between different automatic fingerprint verification algorithms [50]. A performance model is built based on statistics. It can be applied to estimate the uniqueness of the template in classifiers [50].

#### 1.4.4. Performance Prediction System or Performance Prediction Testbed

The afore-mentioned image measures (CFAR, power spectrum signature and probability of edge) are applied in a software which is implemented to validate the performance of some infrared (IR) image-based ATR algorithms [20]. For an imagery automatic target detection (ATD) system, these metrics are also employed in a software tool developed at Los Alamos National Laboratory [64]. A prototype software is developed to reveal the computational error of an ATR system [60,61].

### 1.5. Limitations of the Available Approaches on Performance Evaluation and Performance Prediction for an ATR System

**Figure 1.**
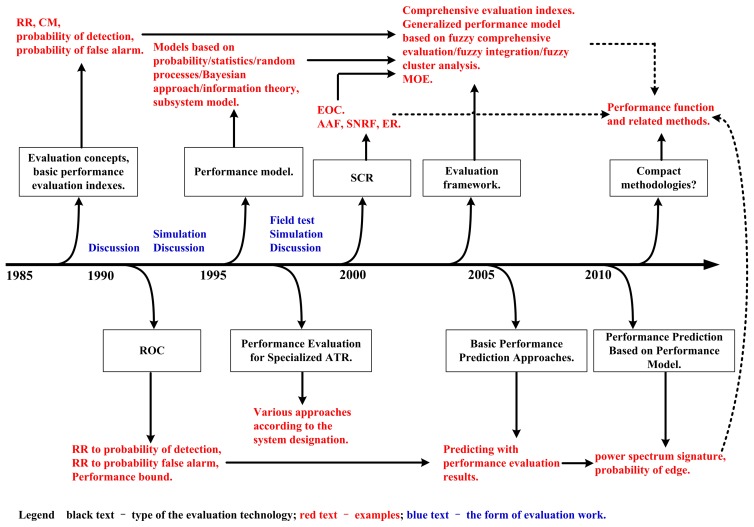
The time-line of the evolution in PE-ATR & PP-ATR.

Based on the materials presented above, the time-line of the evolution in PE-ATR is summarized in Figure 1. In the performance evaluation and the performance prediction work for an ATR system, the aforementioned methods offer choices for us. However, there are still remarkable weaknesses in this area:First of all, in the calculating course, most of the performance evaluation and the performance prediction approaches have not taken the operating condition into account. As a result, the performance evaluation and the performance prediction output may lack of objectiveness [19].

Secondly, the performance evaluation methods available can not work flexibly and no general reference frame has yet been built [22,41]. Furthermore, some of the performance evaluation indexes are too simple to reveal the problem-solving capability of an ATR system [65].

In addition, there are few perfected performance prediction tools that can be used to field test at present [66].

Therefore, in PE-ATR and PP-ATR, sound methodologies that are flexible to the scenario while exercising objectiveness are key topics [3].

### 1.6. Designation Objective of this Work and Its Layout

The contribution of this paper includes: (a) a measure to scale the operating condition for ATR; (b) the definition of performance evaluation indexes; (c) the construction of performance evaluating and performance predicting function. As a result, a novel approach is developed for the performance evaluation work in ATR. Compared to the existing methods, this approach is compacted, scenario adapting and easy to configure. In the evaluation or prediction course, this novel approach takes the operating condition into account, an objective conclusion may be arrived at.

In organizing this paper, the problem and its background are analyzed firstly. The key ideas of this work are explained. These are the main contents in Section 1. The rest of the data is organized as follows:

- The majority of our work concerns the performance evaluation and performance evaluation work in ATR. This is further detailed in Section 2.
  The general idea of this methodology is summarized in Section 2.1.
  In Section 2.2, some similar technics related to ATR is identified and the ATR system's output is classified. The sample size in various experiments is resolved.
  To offer an objective evaluation conclusion, ATR system's condition is scaled in Section 2.3. The proposed index is enlightened by the measures of similarity in pattern recognition.
  In Sections 2.4 and 2.5, the performance evaluation work is implemented with performance evaluation indexes and an evaluation function. The proposed performance evaluation indexes are built based on the probability and mathematical statistics. The most important principles are the tests of statistical hypothesis: the hypothesis test of distribution specialty and the hypothesis test of independence.
  In Section 2.6, the performance predicting is realized with a generalized function. Based on the idea in expert prediction (EP, a branch of machine learning), the proposed performance predicting approach is built.
- To confirm the practicability of this work, experiments are implemented in Section 3. The ATR algorithms setup and the data are explained in Section 3.1. Simulation results and the analysis of them are shown in Sections 3.2–3.4. Comprehensive topics related to this work are discussed in Section 3.5.
- In Section 4, a summary is provided and the future topics are suggested.

In view of the proposed indexes, this work spans a number of scientific disciplines, and there are many references concerning those topics, though the related scientific background has not been presented in the text. However, the scientific background is figured out for each proposed index.

## 2. The Algorithm to Understand, Evaluate, and Predict the Performance of an ATR System

### 2.1. The Idea to Evaluate an ATR System's Performance

Because the ATR system is flexible and many constituent components interact in a complicated way, it is impossible to model an ATR system's output as the function of all the effective factors. A more viable approach (the idea in this work) is to observe the input and the corresponding output, and to determine the comprehensive performance in handling a certain target [26]. In carrying out the theoretics part of this work, we follow the listed steps below.

- The definition of ATR is firstly investigated. The ATR system's output is classified. These are the foundation of the entire work.
- Secondly, an index is proposed to scale the operating condition for recognition. This index can be further utilized in developing the performance evaluation index and performance evaluation function.
- Thirdly, a series of evaluation index is developed. The precision, the robustness and the independence of the recognition output are measured.
- The fourth step is building a performance evaluation function. The proposed evaluation indexes and the operating condition are integrated. A general conclusion may be arrived at with this function.
- The final step is developing an algorithm to predict the ATR system's performance.

**Figure 2.**
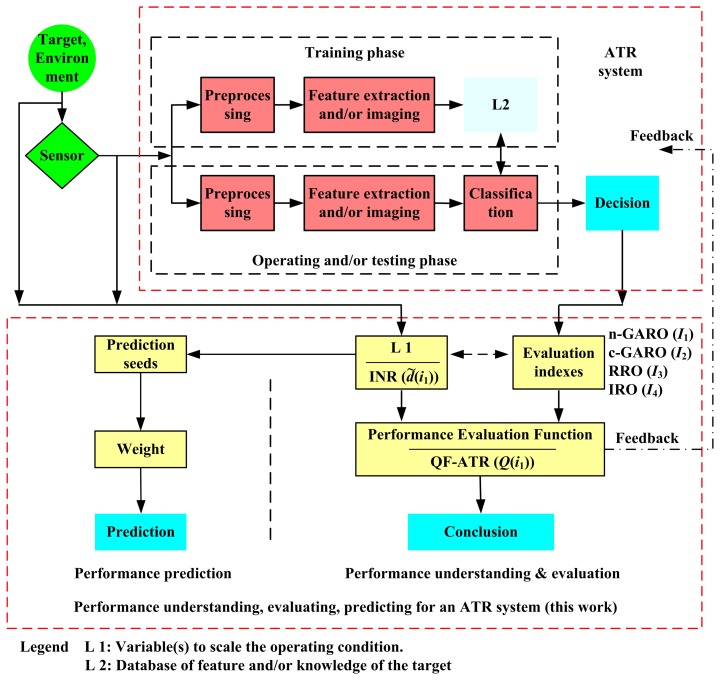
The practical way to evaluate an ATR system's performance.

The idea and the main contribution is shown in Figure 2.

### 2.2. The Definition of Automatic Target Recognition, the Identification of Some Similar Technics, and the Classification of an ATR System's Output

As most researchers will admit, the main component of ATR is a signal processing course which trains the system with information regarding the concerned target in advance. The system can then be used to make decisions on the input signal about the potential target. Usually, its output is used for further decision making or action. Typically, there are three terms relating to this system: “classification”, “recognition” and “identification”. Some scientists have discussed this point [1,3]. Here, ATR is divided between automatic target discrimination (ATD) and automatic target identification (ATI). If the feed signal contains information from the trained target, the processing course is then called ATR. If there is only information from an untrained target in the collected signal, the processing course is called ATD, which, in nature, discriminates the signal as “having no information related to any trained target”. Moreover, if there is no information from any target in the obtained signal, then the processing course is called ATI. This, essentially identifies the signal as “having no information related to any target at all”.

**Figure 3.**
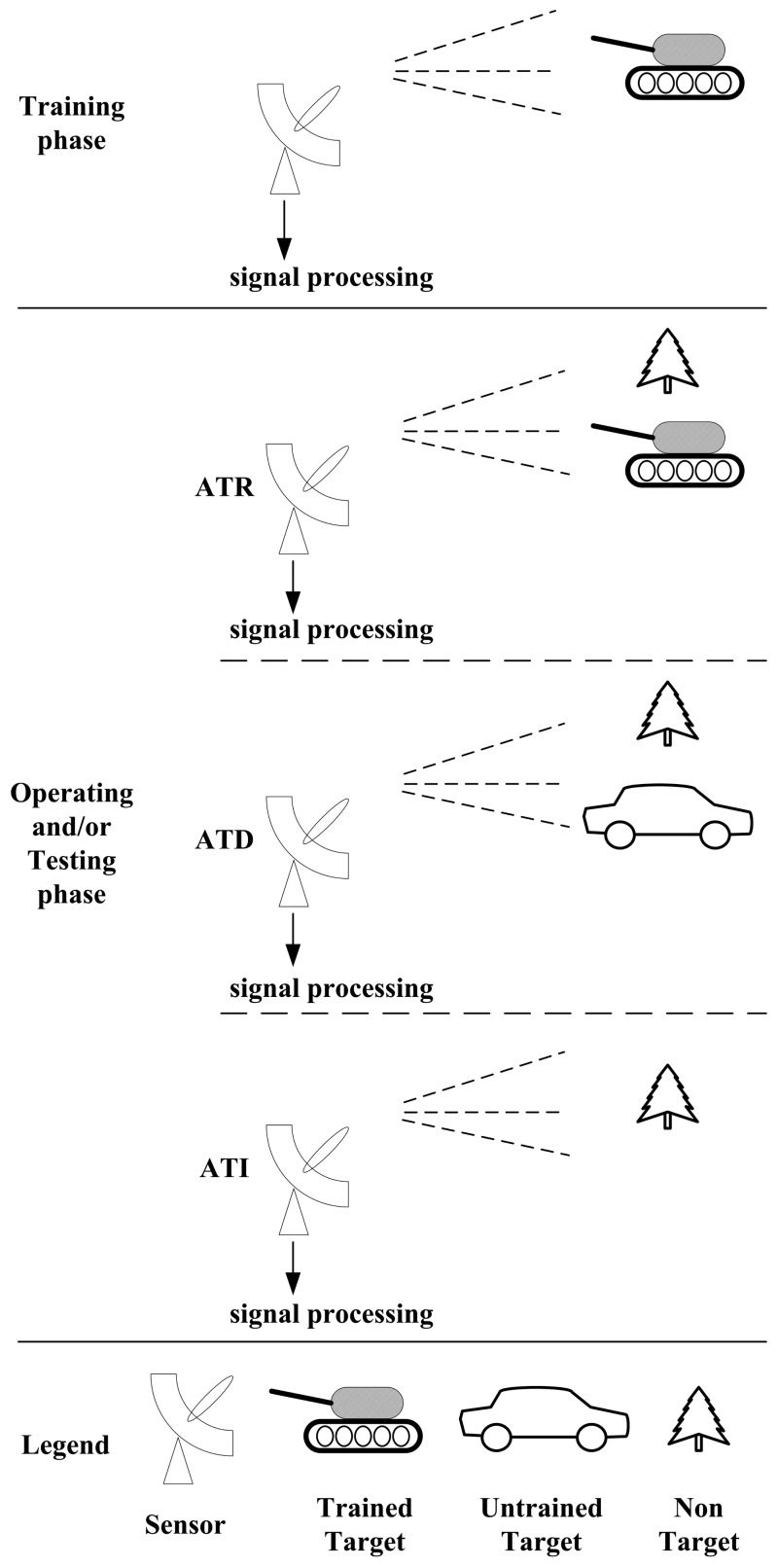
The difference among ATR, ATD and ATI.

The difference of these three technologies related to ATR is shown in Figure 3.

With these preparations, the output of an ATR system can be classified as in Table 1, where the variable *n_ij_* for each category is the corresponding sample size when there are *N* tests in total, $N=\overset{3}{\underset{i=1}{\text{∑}}}\overset{4}{\underset{j=1}{\text{∑}}}n_{ij}$; and [Info.], [R], [D], [I] stand for “information”, “recognition”, “discrimination”, and “identification”, respectively. It can be seen that there are three types of signal fed to the sensor: “target A”, “an untrained target”, and “no target”. So, *i* = 1, 2, 3. There are four types of output of the ATR system. So, *j* = 1, 2, 3, 4. Each false decision in these activities can be classified into a false type, as is shown in Table 1.

The designation of Table 1 is as follows. When the feeding signal containing information from target A, “False [R]” is the name of the decision type that the ATR system's output is another trained target other than target A, “Omitted [R]” is the name of the decision type that the ATR system's output is “cannot figure out the target type, ” and “True [R]” is the name of the decision type that the ATR system's output is target A.

### 2.3. Scaling the Condition for Recognition

In order to judge the ATR systems in an objective way, one must scale the condition for recognition. This is measured by a novel developed quantity called “Innovation for recognition (INR)”, which, through calculating the distance of the samples inside a certain target type and the distance among different target types, indicates the degree of difficulty in recognizing a certain trained target.

Firstly, for the testing samples (testing data) and the training samples (training data), the distance of the target's feature column vector between them is considered.

Suppose there are *t*_1_ different types of training targets in the system, the targets are distinguished by features in *m* dimensions, **x**^(^*^i^*^_1_,^*^i^*^_2_)^ is the feature column vector of the target's testing samples. *i*_1_ shows the serial number of the target. *i*_2_ indicates the serial number of the sample. Here, *i*_1_ = 1, 2, …, *t*_1_, *i*_2_ = 1, 2, …, $S(i_{1}^{\text{w}})$. $S(i_{1}^{\text{w}})$ is the total number of the testing samples, **x̃**^(^*^i^*^_3_,^*^i^*^_4_)^ is the feature column vector of the target's training samples. *i*_3_ shows the serial number of the target. *i*_4_ indicates the serial number of the sample. Here, *i_3_* = 1, 2, …, *t*_1_, *i*_4_ = 1, 2, …, $S(i_{3}^{\text{t}})$. $S(i_{3}^{\text{t}})$ is the total number of the training samples. As a result, for target *i*_1_ and target *i*_3_,

(1) $$\delta ({\text{x}}^{\left(i_{1},i_{2}\right)},{\tilde{\text{x}}}^{\left(i_{3},i_{4}\right)})={\left[{\left({\text{x}}^{\left(i_{1},i_{2}\right)}-{\tilde{\text{x}}}^{\left(i_{3},i_{4}\right)}\right)}^{\text{T}}({\text{x}}^{\left(i_{1},i_{2}\right)}-{\tilde{\text{x}}}^{\left(i_{3},i_{4}\right)})\right]}^{1/2}$$

is the distance of the feature column vector from these two sets of samples.

Then, the INR of target *i*_1_ can be solved by:

(2) $$\begin{array}{c} \tilde{d}(i_{1})=\frac{1}{\left(t_{1}-1\right)}\cdot \frac{\overset{S(i_{1}^{\text{w}})}{\underset{i_{2}=1}{\text{∑}}}\left(\overset{t_{1},i_{3}\neq i_{1}}{\underset{i_{3}=1}{\text{∑}}}\overset{S(i_{3}^{\text{t}})}{\underset{i_{4}=1}{\text{∑}}}\delta_{1}(\cdot )\right)}{\left[\overset{t_{1}}{\underset{i_{1}=1}{\text{∑}}}S(i_{1}^{\text{w}})\right]\;\left[\overset{t_{1},i_{3}\neq i_{1}}{\underset{i_{3}=1}{\text{∑}}}S(i_{3}^{\text{t}})\right]}\cdot \frac{1}{\frac{1}{S(i_{1}^{\text{w}})S(i_{1}^{\text{t}})}\cdot \overset{S(i_{1}^{\text{w}})}{\underset{i_{2}=1}{\text{∑}}}\left(\overset{S(i_{1}^{\text{t}})}{\underset{i_{4}=1}{\text{∑}}}\delta_{2}(\cdot )\right)}\\ =\frac{S(i_{1}^{\text{w}})S(i_{1}^{\text{t}})\overset{S(i_{1}^{\text{w}})}{\underset{i_{2}=1}{\text{∑}}}\left(\overset{t_{1},i_{3}\neq i_{1}}{\underset{i_{3}=1}{\text{∑}}}\overset{S(i_{3}^{\text{t}})}{\underset{i_{4}=1}{\text{∑}}}\delta_{1}(\cdot )\right)}{(t_{1}-1)\left[\overset{t_{1}}{\underset{i_{1}=1}{\text{∑}}}S(i_{1}^{\text{w}})\right]\;\left[\overset{t_{1},i_{3}\neq i_{1}}{\underset{i_{3}=1}{\text{∑}}}S(i_{3}^{\text{t}})\right]\;\overset{S(i_{1}^{\text{w}})}{\underset{i_{2}=1}{\text{∑}}}\left(\overset{S(i_{1}^{\text{t}})}{\underset{i_{4}=1}{\text{∑}}}\delta_{2}(\cdot )\right)} \end{array}$$

where $\delta_{1}(\cdot )=\delta ({\text{x}}^{\left(i_{1},i_{2}\right)},{\tilde{\text{x}}}^{\left(i_{3},1_{4}\right)}),\delta_{2}(\cdot )=\delta ({\text{x}}^{\left(i_{1},i_{2}\right)},{\tilde{\text{x}}}^{\left(i_{1},i_{4}\right)})$. In Equation (2),

(3) $$\frac{\overset{S(i_{1}^{\text{w}})}{\underset{i_{2}=1}{\text{∑}}}\left(\overset{t_{1},i_{3}\neq i_{1}}{\underset{i_{3}=1}{\text{∑}}}\overset{S(i_{3}^{\text{t}})}{\underset{i_{4}=1}{\text{∑}}}\delta (\cdot )\right)}{\left[\overset{t_{1}}{\underset{i_{1}=1}{\text{∑}}}S(i_{1}^{\text{w}})\right]\;\left[\overset{t_{1},i_{3}\neq i_{1}}{\underset{i_{3}=1}{\text{∑}}}S(i_{3}^{\text{t}})\right]}$$

indicates the distance between the feature column vectors from (a) the testing samples of the training target *i*_1_, and (b) the training samples of all the training targets except for target *i*_1_. Here,

(4) $$\frac{1}{S(i_{1}^{\text{w}})S(i_{1}^{\text{t}})}\cdot \overset{S(i_{1}^{\text{w}})}{\underset{i_{2}=1}{\text{∑}}}\left(\overset{S(i_{1}^{\text{t}})}{\underset{i_{4}=1}{\text{∑}}}\delta (\cdot )\right)$$

shows the distance between the feature column vectors from (a) the testing samples of the training target *i*_1_, and (b) the training samples of the target *i*_1_. $\frac{1}{\left(t_{1}-1\right)}$ brings in an average among *t*_1_*–* 1 targets. The normalized form of INR is used generally,

(5) $$d(i_{1})=\tilde{d}(i_{1})/d_{0}$$

where $d_{0}=\max\left(\tilde{d}(i_{1})\right)$ is the maximum value for all possible operating conditions. For a certain ATR system handling a certain target, the lower the INR, the more difficult it is to perform the recognition task.

In building the INR index, the related principle is the knowledge of feature extraction in pattern recognition, as is detailed in many literatures [67].

### 2.4. Performance Evaluation Indexes

For a practical ATR system, an accurate and robust output is overwhelmingly welcomed. It is important that the result should be independent to the run condition, or at least, should be influenced as little as possible. The following capacities are concerned:

(a) The general approach of the recognition output (GARO). GARO weighs the recognition output, on the basis of whether or not it comes up with the desired level on correct decisions. Suppose the sample size in Table 1 fulfills the requirements in hypothesis testing. There are two schemes for GARO: naked GARO (n-GARO) and GARO with cost (c-GARO), denoted by *I*_1_ and *I*_2_ respectively,
  (6) $$I_{1}=\frac{n_{11}}{\overset{4}{\underset{j=2}{\text{∑}}}n_{1j}}\cdot \frac{n_{23}}{\overset{4,j\neq 3}{\underset{j=1}{\text{∑}}}n_{2j}}\cdot \frac{n_{34}}{\overset{3}{\underset{j=1}{\text{∑}}}n_{3j}}$$
  (7) $$I_{2}=\frac{\omega_{11}n_{11}}{\overset{4}{\underset{j=2}{\text{∑}}}\omega_{1j}n_{1j}}\cdot \frac{\omega_{23}n_{23}}{\overset{4,j\neq 3}{\underset{j=1}{\text{∑}}}\omega_{2j}n_{2j}}\cdot \frac{\omega_{24}n_{34}}{\overset{3}{\underset{j=1}{\text{∑}}}\omega_{3j}n_{3j}}$$
  Here, *ω_ij_* ≥ 1, *i*, *j* = 1, 2, 3, 4 are the assigned value of cost, usually, *ω*_11_ = *ω*_23_ = *ω*_34_ = 1. The cost in c-GARO is introduced to distinguish the risk of different types of decisions. These costs are empirically set according to the scenario.
  If any fraction of *I*_1_ and/or *I*_2_ fall(s) into the 0/0 form, it is then set to 1.
  The n-GARO is introduced with the knowledge of “summary measures” in statistics [68,69], while the c-GARO is found with the knowledge of “summary measures” in statistics and “numeric analysis” in engineering mathematics [68,70].
(b) The robustness of the recognition output (RRO). RRO checks whether the operating output samples have the same distribution as that in the training course. RRO is revealed by the distribution specialty of GARO, through a rank-sum test. The related knowledge is “hypothesis testing” in statistics [68,69].
  Suppose that there are *n*_1_ samples of n-GARO in the training course, while there are *n*_2_ samples of n-GARO in the testing course, all these samples are obtained under the same INR level. That is to say, within the same INR confidence interval. The Wilcoxon rank-sum test is applied [68]. Let *R*_1_ stand for the rank summation of the training samples, then,
  (8) $$I_{3}={\left\{1+\left|\ln\;\left[\frac{2R_{1}-n_{1}(n_{1}+1)}{n_{1}(n_{1}+2n_{2}+1)-2R_{1}}\right]\right|\right\}}^{-1}$$
  is the normalized RRO. It shows whether the two concerned sample sets are subject to a uniform distribution, the idealized value of *I_3_* is 1. Proof of this point can be found in [68,69].
  The RRO with cost has not been touched here, as it can be arrived at in a similar manner.
(c) The independence of the recognition output to condition (IRO). Through the hypothesis test of independence, IRO estimates the independence of the recognition output to condition. Here, the hypothesis test of independence shows the influence (or impact) of the testing condition on the ATR system's performance. The related knowledge is “hypothesis testing” in statistics [68,69].

The two sets involved in the test are INR and n-GARO. There are *s*_1_ subclasses in INR and *s*_2_ subclasses in n-GARO, *P*(INR = *i*, n-GARO = *j*) = *p_ij_*, ∀*i* ∈ [1, *s*_1_], ∀*j* ∈ [1, *s*_2_]. The population (INR, n-GARO) has a sample size *m*. *m_ij_* is the sample size when INR is in its *i^th^* subclass and n-GARO is in its *j^th^* subclass, *m_i_*. is the sample size when INR is in its *i^th^* subclass and all n-GARO subclasses. *p_i_*_·_ = *m_i_*_·_/*m*_·_*m*_·_*_j_* and *p*_·_*_j_* have the similar meaning for n-GARO. Let:

(9) $$\chi^{2}=\overset{s_{1}}{\underset{i=1}{\text{∑}}}\overset{s_{2}}{\underset{j=1}{\text{∑}}}\frac{{\left(m_{ij}-mp_{i\cdot }\;\cdot \;p_{\cdot j}\right)}^{2}}{mp_{i\cdot }\;\cdot \;p_{\cdot j}}=\overset{s_{1}}{\underset{i=1}{\text{∑}}}\overset{s_{2}}{\underset{j=1}{\text{∑}}}\frac{{\left(m_{ij}-m_{i\cdot }m_{\cdot j}/m\right)}^{2}}{m_{i\cdot }m_{\cdot j}/m}$$

stand for the test statistic, the threshold is *η*, then, IRO is arrived at by:

(10) $$I_{4}={\left[1+\frac{1}{\Lambda }\left|\text{ln}\;(\chi^{2}/\eta )\right|\right]}^{-1}$$

where Λ is the variation range of *d*(*i*_1_), the idealized value of *I*_4_ is 1.

Further materials related to the hypothesis test of independence can be found in [68,71].

### 2.5. The Way to Understand and Evaluate the Performance of an ATR System

On the basis of the previous work, the performance of an ATR system can be interpreted and evaluated in two ways. One is to list the value of INR and the corresponding evaluation indexes. This can be easily realized, but the result can not be understood well by people outside of this field. Another way is to introduce a comprehensive function from these parameters, namely, the quality factor of the ATR system (QF-ATR) in attacking target *i*_1_,

(11) $$Q(i_{1})=(I_{1}(i_{1})I_{3}(i_{1})I_{4}(i_{1}))/d(i_{1})$$

*i*_1_ = 1, 2, …, *t*_1_, through applying the Monte Carlo test, the final comprehensive comment may be obtained. Here, *Q*(*i*_1_) is the expression of QF-ATR in the calculating course. It should be noted that for *I*_4_(*i*_1_) in calculating QF-ATR, *d*(*i*_1_) is the mean value of its variation interval. When recognizing a certain target under a certain situation, the larger the QF-ATR is, the better performance the system maintains.

In a similar way, the QF-ATR with cost is resolved.

The QF-ATR index is introduced with the knowledge of “summary measures” in statistics and “numeric analysis” in engineering mathematics [68,70].

### 2.6. Predicting the Performance of an ATR System

Performance prediction work can be classified into three situational categories: (a) forecasting the performance for a repeated test with a familiar system and target; (b) predicting the performance for a tested system on a newly trained target; (c) figuring out the capability for a new ATR system on a familiar or a novel target. As an example, *I*_1_ is chosen as the performance index to be predicted.

First, to estimate the performance in managing a trained target *i*_1_ for a repeated test with INR equaling *d_j_*(*i*_1_), the test records of this target, with consistent INR, are taken from the database. These records are the seeds for forecasting work. A term imposed on the newly born set is that, its sample size should be no less than the requirements originating from the corresponding hypothesis testing. This term is effective here as well as in the following cases.

Another mission, is that to estimate the performance for the first test in coping with a newly trained target *i_n_*. In order to proceed with the forecasting work, the database of training output is consulted. While the operating conditions are much more substantial, it is supposed that the target's INR states fall into *d_j_*(*i_n_*), *j* = 1, 2, …, *J* in the training course. For predicting the performance in a certain state *d_m_*(*i_n_*), *m* = 1, 2, …, *M*, the training record, whose INR is *d_m_*(*i_n_*) ± *o*, is taken out. These records are the seeds for performing the prediction work. Here, *o* is a reasonable tiny quantity in practice. For example, *o* = 0.05 × *d_j_*(*i_n_*).

The third one, but not the least important, is that to predict the performance for a newly developed ATR system, people may consult the systems with similar approaches in processing same or similar targets. The procedures are not duplicated as they are similar to those in the previous situations. One should be aware that even for the same target within a uniform environment, the INR may be different in different systems.

Once the preparation has been completed, a novel developed prediction methodology, referred to as “context-probability (CP)”, is applied. CP is useful for estimation and forecasting work in complicated systems such as an ATR system, where there are many different variables interacting in a complex fashion that can not be figured out in clear expressions. In addition, the system may provide increasingly accurate and robust results by incorporating historical data into the calculations. So, the new measure should take into account both sequential information and probability. The procedures are:

(a) Collecting the seeds for prediction according to their sequence, here, *I*_1_(l), *I*_1_(2), …, *I*_1_(*k*) are harvested;
(b) Calculating the context weight for the collected seeds, $\omega_{1}(m)=e^{\left(\frac{-m}{k}\right)}/\overset{k}{\underset{j=1}{\text{∑}}}e^{\left(\frac{-j}{k}\right)}$, *m* = 1, 2, …, *k*;
(c) Calculating the probability weight for the collected seeds, $\omega_{2}(m)=\Delta (m)/\overset{k}{\underset{j=1}{\text{∑}}}\Delta (j)$, *m* = 1, 2, …, *k*; where Δ(*m*) and Δ(*j*) subject to the identical form: $\Delta (\mu )=\frac{\overset{k}{\underset{t=1}{\text{∑}}}\left|I_{1}(t)-\left(\overset{k}{\underset{r=1}{\text{∑}}}I_{1}(r)/k)\right)\right|}{\left|I_{1}(\mu )-\left(\overset{k}{\underset{r=1}{\text{∑}}}I_{1}(r)/k)\right)\right|},\mu =1,2,\ldots ,k;$
(d) Calculating the general weight for the collected seeds, $\omega (m)=\frac{\omega_{1}(m)\omega_{2}(m)}{\overset{k}{\underset{j=1}{\text{∑}}}\omega_{1}(j)\omega_{2}(j)}$; *m* = 1, 2, …, *k*;
(e) Releasing the forecasting result for the system, $I_{1}(k+1)=\overset{k}{\underset{j=1}{\text{∑}}}\omega (j)I_{1}(j)$.

It is clear that for this kind of weighted average prediction, there is a group of choices for the weight average strategy. The above-mentioned way is one of them. The principal requirements for the weight average strategy are: (a) the fresher the data point, the larger the weight is; (b) the less distance between the data point and the mean value, the larger the weight is; (c) the final weight vector should be a normalized one.

As mentioned before, when one takes the knowledge of probability, statistics, and weighted average prediction into mind, a kind of performance prediction method is realized. Aside from this predicting method, one can forecast an ATR system's performance by using a machine learning facility called expert prediction [72], or with a data processing technology called bootstrapping [73]. In most situations, this method outperforms the others in that both the sequence and the probability are considered.

**Figure 4.**
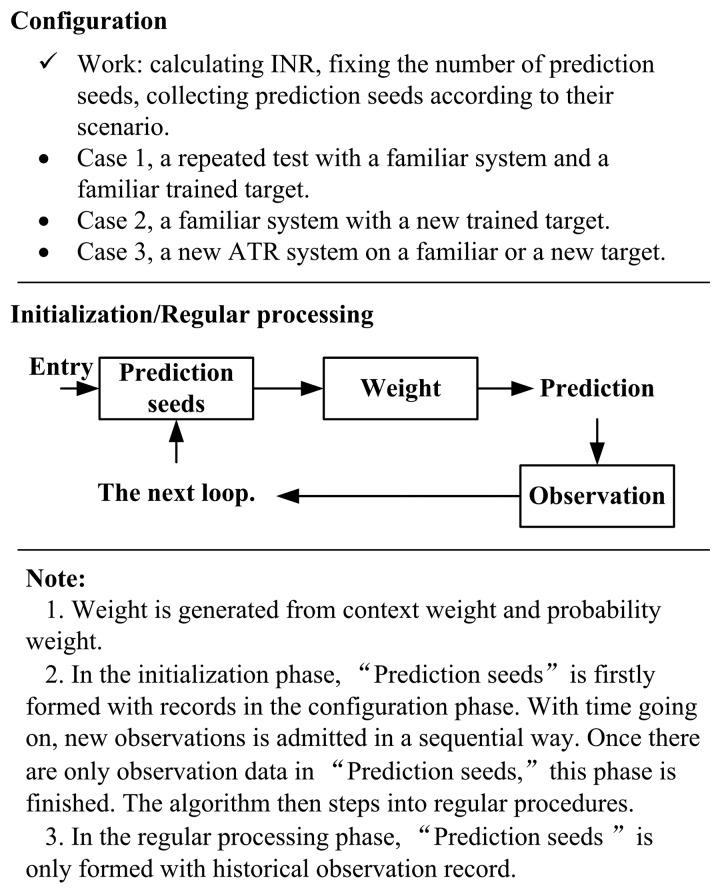
The configuration steps of the proposed prediction algorithm.

The flow diagram of the prediction algorithm is shown in Figure 4.

### 2.7. Summary of the Proposed Methodologies

As we have witnessed, the compilation of this work has thus far comprised of the performance evaluation measure for an ATR system, the performance prediction method for an ATR system, and a quantity to scale the operating condition is developed. The proposed methodologies are collected in Table 2. The relation among these performance indexes is shown in Figure 2. In Table 2, “SCR” means “Scaling the Condition for Recognition”.

## 3. Experiments

To validate this novel methodology, a series of simulations have been undertaken. A sampling of results follows. Before starting the discussion of the simulation, we should emphasize that the experiments here are: (a) to check whether the evaluation conclusion is in accordance with the performance inference; (b) to check whether the performance prediction output is proper compared to the practical performance; and (c) to validate whether the methodology can be applied to a variety range of ATR systems. Therefore, when performing experiments, there are 3 kind of ATR systems being tested. The capability of the proposed methods to be applied in various ATR systems is thus validated. Moreover, two similar ATR systems are considered. This is to check the ability of distinguishing the performance of similar ATR systems in similar scenarios.

### 3.1. The ATR Algorithms Setup and the Data

The proposed methodology in this work can be applied to all ATR systems and algorithms. However, the algorithms under consideration in the experiments are limited. There are 4 ATR algorithms taken into account: a SAR ATR method based on a global scattering center model [74], an improved approach for target discrimination in high-resolution SAR images [75], and an electrocardiograph (ECG) waveform recognition algorithm based on sparse decomposition and neural network (NN) [76]. They are named as Sys1, Sys2, and Sys3A respectively; a modified electroencephalograph (EEG) signal recognition measure based on empirical mode decomposition (EMD) and autoregression (AR), namely, Sys3B, is developed and validated to compare the performance results as in Sys3A.

Sys1 is configured according to [74] (recognizing targets I, II, and III, and is referred to as recognizing target 1, 2, and 3 in this work). Sys2 is implemented from [75] (recognizing target 6, 7, and 9, and is referred to as recognizing T6, T7, and T9 throughout this work). Sys3A is accomplished in conformity to [76] (recognizing P Pulse and T Pulse in this work). The EMD subsystem of Sys3B in feature extraction is directly implemented with respect to the EMD subsystem in [77]. The classifier in Sys3B is realized according to the classifier in [78]. The other subsystems in Sys3B and Sys3A are identical.

Sys1 and Sys2 are trained and tested with the data from [74,75], respectively; while Sys3A is trained and tested with the data from PhysioNet [79]. Sys3B is applied to the same data as in Sys3A.

The EEG data of University of California Irvine (UCI) arises from a large study to examine EEG correlates of genetic predisposition to alcoholism [80]. It contains measurements from 64 electrodes (medical sensors) placed on the scalp sampled at 256 Hz. Both the training portion and the test portion of the large data set are applied. The ECG data from PhysioNet applied are ECG [Class 1; core] long-term ST database.

### 3.2. Selected Simulation Results and Analysis

#### 3.2.1. Partial Results (Performance Evaluation and Performance Prediction) and Analysis

Some of the performance evaluation results are given in Table 3, while the performance forecasting results and validation there of are shown in Table 4. The “PE” in these tables means “performance evaluation.” For each record of the performance evaluation indexes, the original sensing and recognizing sample size is 150 times. As for each record in Tables 3 and 4, it is obtained using the Monte Carlo test with 50 runs. The principle of performance model based on fuzzy integration (PM-FI) is detailed in [9]. The performance indexes (*I*_1_/*I*_3_/*I*_4_) are considered in PM-FI. The weight in these three indexes are all set as 1.

In Table 3, several interesting conclusions can be drawn. First, the recommended methodologies can offer well-founded judgment for the system, as long as the operating condition is varying. Secondly, the QF-ATR consider the performance not only with the output, but also with the operating condition. For example, the *I*_1_ level of Sys2 is much better than that of Sys1. At the same time, the value of *I_3_* and *I*_4_ from these two systems are almost similar. It is unfortunate, that QF-ATR of Sys2 is about half of Sys1. The reason lies in the condition, as is indicated by INR. Third, this facility can clearly discriminate between systems when they handle identical targets under identical conditions. The evaluation results from Sys3A and Sys3B support this point. It is sure that EMD and AR methods maintain less relevance with the condition than sparse decomposition and neural network methods. The figures are in accordance with the inference.

In Table 4, the gap between the forecasting result and the actual output is slim. However, we should pay attention to the fact that each record is the mean value of 50 original performance prediction runs. The prediction error at each prediction step is still clear, as is shown by figures in the following subsections. The result in Table 4 is exciting. It is obvious that the prediction error of QF-ATR is much stronger than the other indexes. This stems from that QF-ATR is the function of the other variables. All the error will be collected into QF-ATR.

It may seem unusual that the QF-ATR can not strictly subject itself to Equation (11) with the listed *I*_1_/*I*_3_/*I*_4_ and INR. This stems from the fact that all indexes in Tables 3 and 4 are processed individually through the Monte Carlo test. The data has been derived individually from the mean value from each 50 run test. The performance prediction is performed using CP only.

Because the scenarios are not complicated, the prediction results of RR have high precision.

#### 3.2.2. Performance Evaluation with ROC Method and Analysis

**Figure 5.**
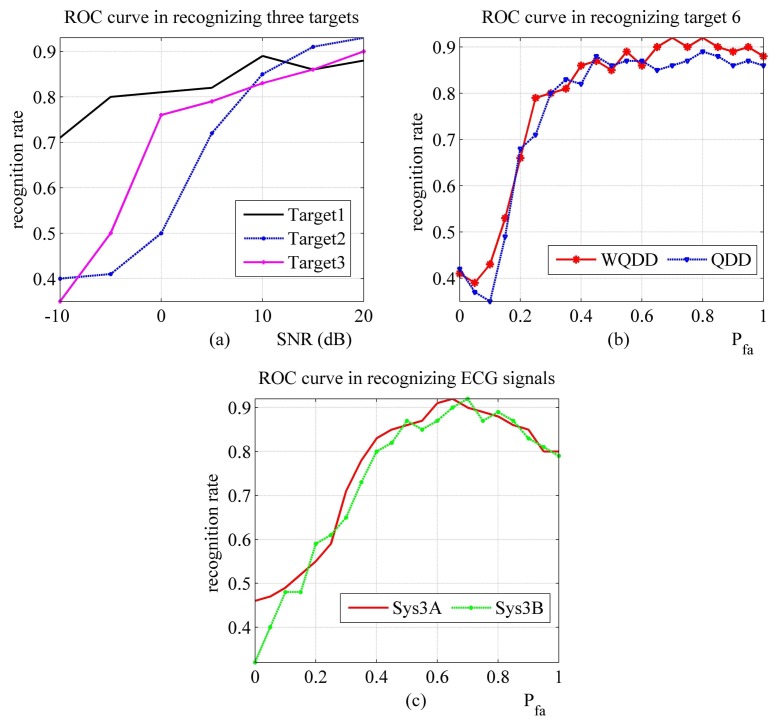
Performance evaluation results of the mentioned ATR systems with ROC approach. (**a**) The recognition rate of Sys1 when SNR is changing; (**b**) The recognition rate of Sys2 when false alarm setting is changing; (**c**) The recognition rate of Sys3A and Sys3B when false alarm setting is changing.

The evaluation results with ROC method are presented in Figure 5. Here, QDD is “quadratic distance discriminator”. WQDD is “weighted quadratic distance discriminator”. For Sys2, Sys3A and Sys3B in Figure 5, it may seem unusual that the RR is little decreasing while *P*_FA_ is greater than a certain value and growing. This stems from the fact that the clutter is too heavy to be effectively processed in those scenarios.

#### 3.2.3. Performance Evaluation with “Confusion Matrix” Method

The evaluation results of confusion matrix method are shown in Tables 5 and 6. In Table 5, “T1” means “Target 1”. The other targets are with the similar name. Here, the settings of the targets for Sys1 are: signal to noise ratio (SNR) is 10 dB, elevation is 10° and the result is arrived at with 500 Monte Carlo simulations [74]. The result of Sys2 is “Experiment and analysis od data provided by the Institute of Electronics, Chinese Academy of Sciences” [75]. In Table 6, “P Pulse” and “T Pulse” are different waveforms which have implications in medical science.

### 3.3. More Simulation Results With Brief Analysis

To clearly show the capability of the methodology, a mere fraction of the simulation results is presented.

**Figure 6.**
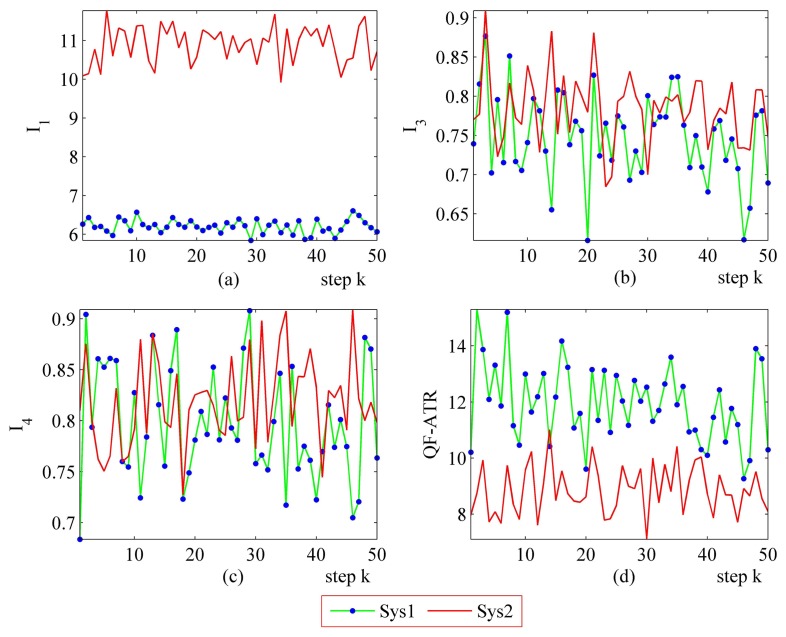
Detailed performance evaluation results of Sys1 and Sys2 for 50 runs, with INR_Sys1 = 0.31, INR_Sys2 = 0.79. (**a**) Detailed results of *I*_1_; (**b**) Detailed results of *h*; (**c**) Detailed results of *I*_4_; (d) Detailed results of *Q*.

The primary setting of the performance evaluation experiments has been collected in Table 7, where “Figure 6, 0.31” means the INR in Figure 6 for the corresponding system is 0.31. The remaining items follow this rule.

The primary setting of the performance prediction simulations has been collected in Table 8, where “Figure 8, 12” means the number of prediction seeds in Figure 8 is 12. The remaining figures follow this rule.

#### 3.3.1. More Performance Evaluation Results on Sys1 and Sys2

**Figure 7.**
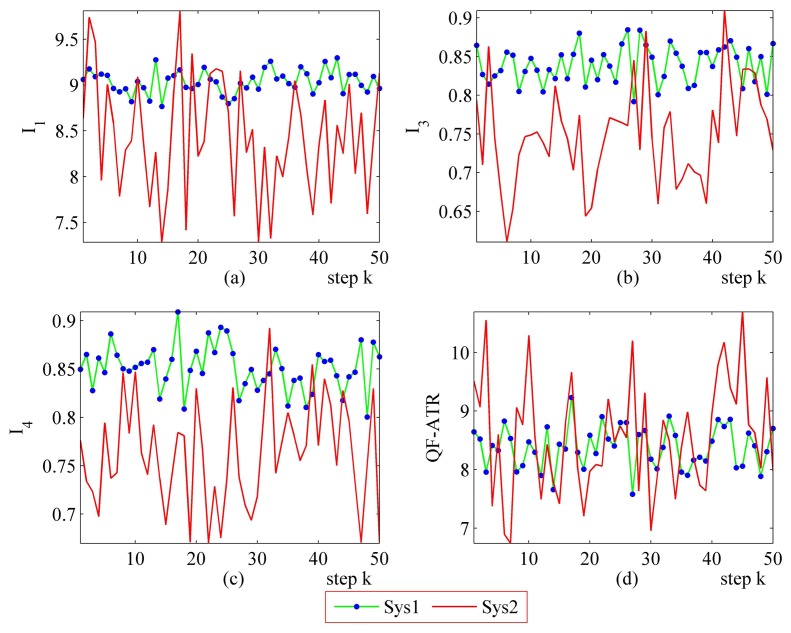
Detailed performance evaluation results of Sys1 and Sys2 for 50 runs, with INR_Sys1 = 0.77; INR_Sys2 = 0.56. (**a**) Detailed results of *I*_1_; (**b**) Detailed results of *I*_3_; (**c**) Detailed results of *I*_4_; (**d**) Detailed results of *Q*.

The step-by-step performance evaluation results are presented in Figures 6 and 7. As demonstrated in Figures 6 and 7, even for a certain ATR system regarding a certain target under a certain condition, the performance shakes. However, the difference exists in the shaking range between different systems.

The upper-left part (Figures 6 and 7) suggests that the *I*_1_ of Sys1 is much more robust than the *I*_1_ of Sys2. For the *I*_3_ and the *I*_4_, Sys1 and Sys2 are similar in the first scenario. Moreover, there is a modest difference in the *I*_3_ and the *I*_4_ from Sys1 and Sys2 in the second scenario. One should be aware that each data point in performance evaluation is arrived at from *N* ATR tests in practice, as is shown in Table 1, as well as subjecting to Equations (6), (8), (10) and (11).

In Figure 6, it may seem unusual that the *I*_1_ of Sys2 is much better than those of Sys1, while the QF-ATR of Sys1 overwhelms those of Sys2. The reason lies in the difference of INR, which shows that the recognition condition is much worse for Sys1 than it is in Sys2.

As presented in these data, the performance of Sys1 is more robust than Sys2 in these two scenarios.

#### 3.3.2. More Performance Prediction Results on Sys1 and Sys2

**Figure 8.**
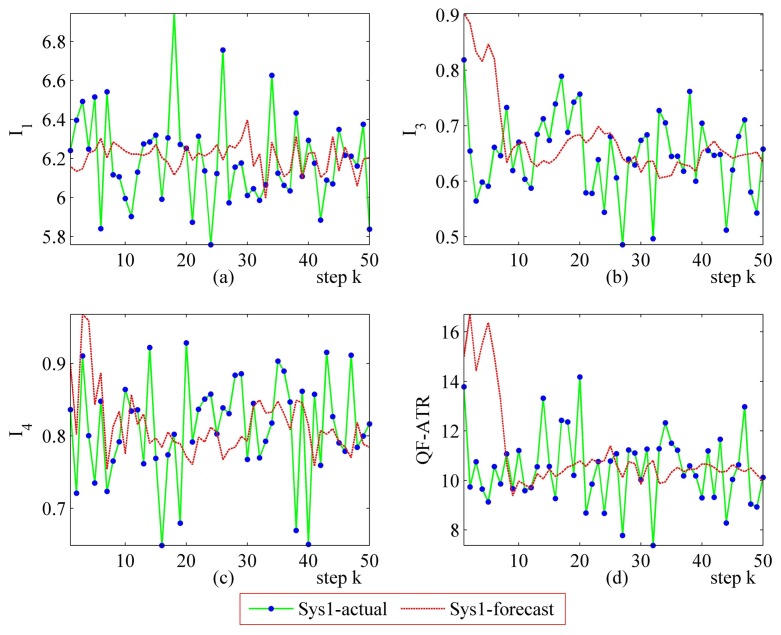
Detailed performance prediction results of Sys1 for 50 runs (INR = 0.31, the number of prediction seeds is 12). (**a**) Predicted and actual *I*_1_; (**b**) Predicted and actual *I*_3_; (**c**) Predicted and actual *I*_4_; (**d**) Predicted and actual *Q*.

**Figure 9.**
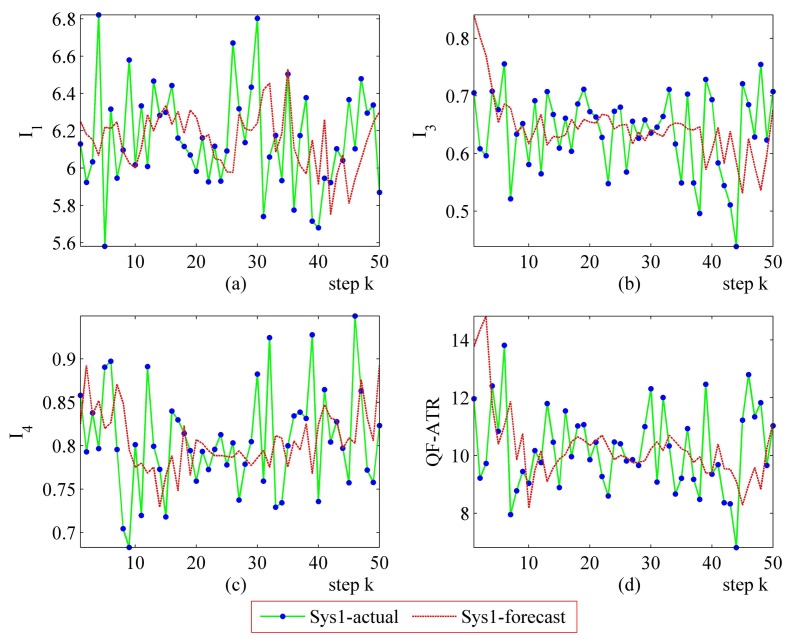
Detailed performance prediction results of Sys1 for 50 runs (INR = 0.31, the number of prediction seeds is 6). (**a**) Predicted and actual *I*_1_; (**b**) Predicted and actual *I*_3_; (**c**) Predicted and actual *I*_4_; (d) Predicted and actual *Q*.

**Figure 10.**
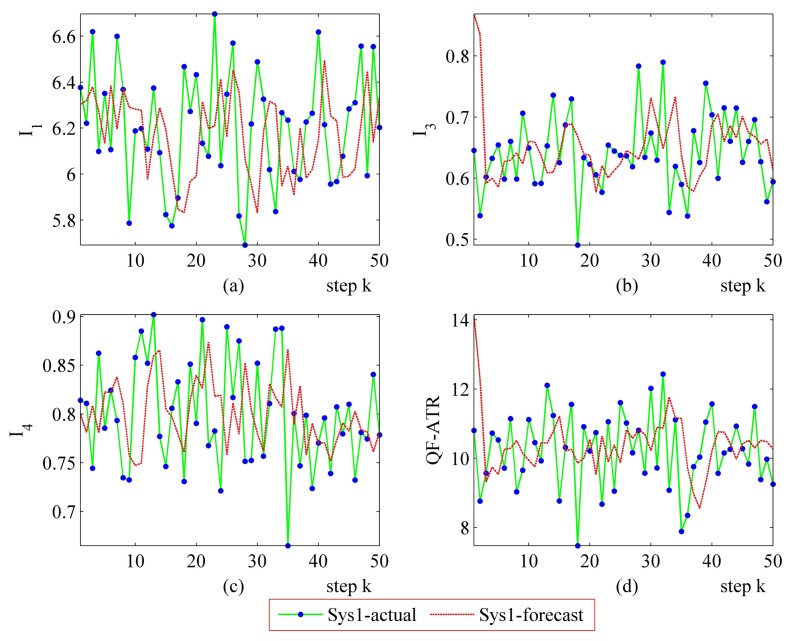
Detailed performance prediction results of Sys1 for 50 runs (INR = 0.31, the number of prediction seeds is 3). (**a**) Predicted and actual *I*_1_; (**b**) Predicted and actual *I*_3_; (**c**) Predicted and actual *I*_4_; (**d**) Predicted and actual *Q*.

**Figure 11.**
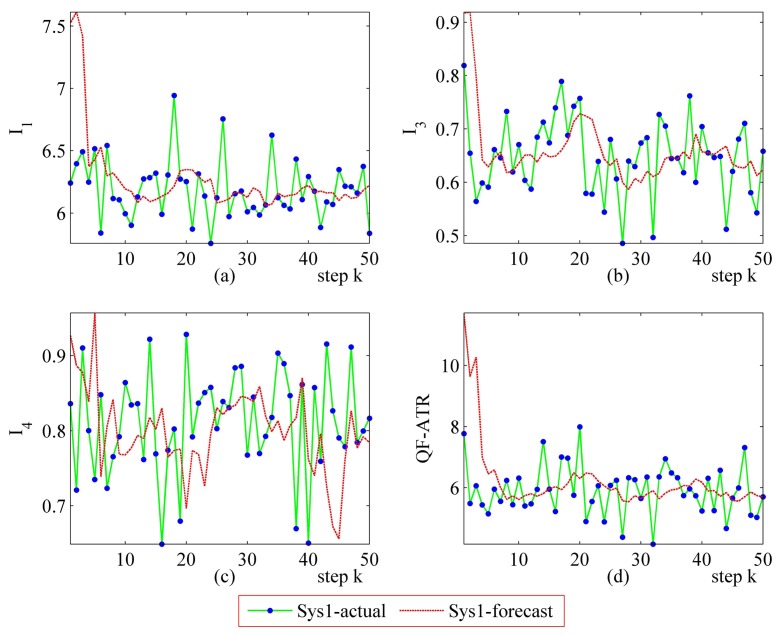
Detailed performance prediction results of Sys1 for 50 runs (INR = 0.55, the number of prediction seeds is 6). (**a**) Predicted and actual *I*_1_; (**b**) Predicted and actual *I*_3_; (**c**) Predicted and actual *I*_4_; (**d**) Predicted and actual *Q*.

**Figure 12.**
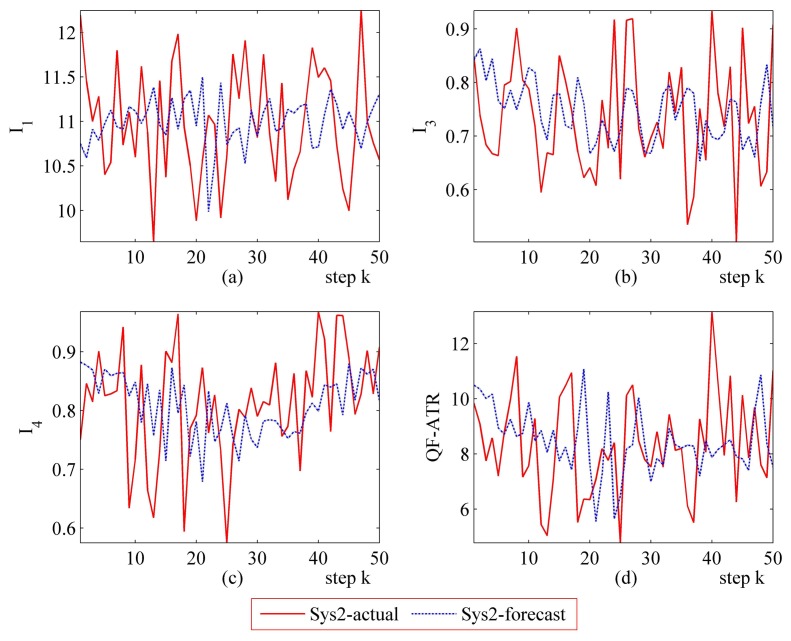
Detailed performance prediction results of Sys2 for 50 runs (INR = 0.79, the number of prediction seeds is 12). (**a**) Predicted and actual *I*_1_; (**b**) Predicted and actual *I*_3_; (**c**) Predicted and actual *I*_4_; (**d**) Predicted and actual *Q*.

**Figure 13.**
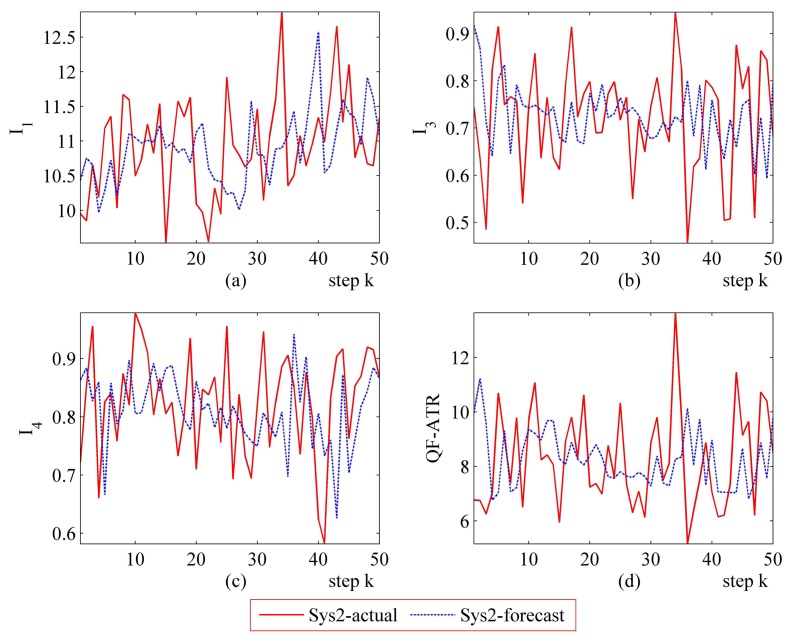
Detailed performance prediction results of Sys2 for 50 runs (INR = 0.79, the number of prediction seeds is 6). (**a**) Predicted and actual *I*_1_; (**b**) Predicted and actual *I*_3_; (**c**) Predicted and actual *I*_4_; (**d**) Predicted and actual *Q*.

**Figure 14.**
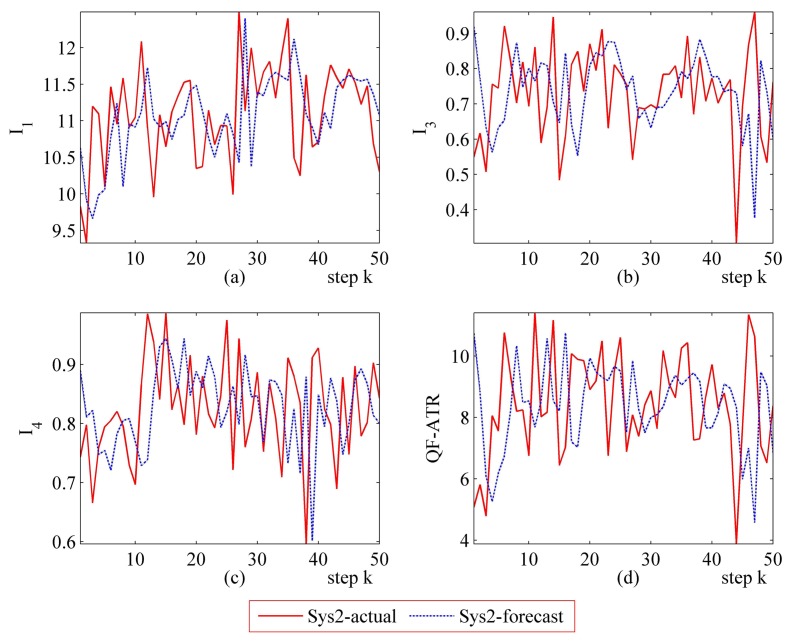
Detailed performance prediction results of Sys2 for 50 runs (INR = 0.79, the number of prediction seeds is 3). (**a**) Predicted and actual *I*_1_; (**b**) Predicted and actual *I*_3_; (**c**) Predicted and actual *I*_4_; (**d**) Predicted and actual *Q*.

**Figure 15.**
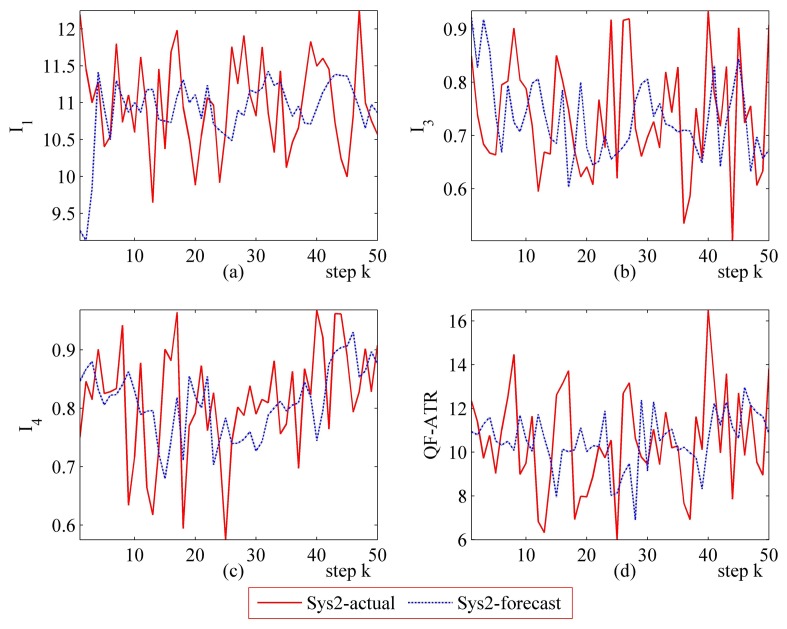
Detailed performance prediction results of Sys2 for 50 runs (INR = 0.63, the number of prediction seeds is 6). (**a**) Predicted and actual *I*_1_; (**b**) Predicted and actual *I*_3_; (**c**) Predicted and actual *I*_4_; (**d**) Predicted and actual *Q*.

Detailed performance prediction results of the above-mentioned Sys1 and Sys2 are given accordingly (Figures 8, 9, 10, 11, 12, 13, 14 and 15). It can be seen that the performance prediction algorithm developed in this work is able to forecast the performance of an ATR system. One should note that each predicted data point here and thereafter is arrived at from a different number of prediction seeds (shown in Table 8), and subjects to the prediction procedures. The actual output is also obtained from *N* tests (shown in Table 1).

From Figures 8, 9, 10, Figures 12, 13, 14, one may know that, for a given ATR system under a certain condition, the fewer the prediction seeds, the more flexible of the prediction ability In most occasions, the especially poor match of the prediction results exists in the initial part. As the prediction continues, the error turns to decline.

#### 3.3.3. Further Performance Evaluation Results on Sys3A and Sys3B

**Figure 16.**
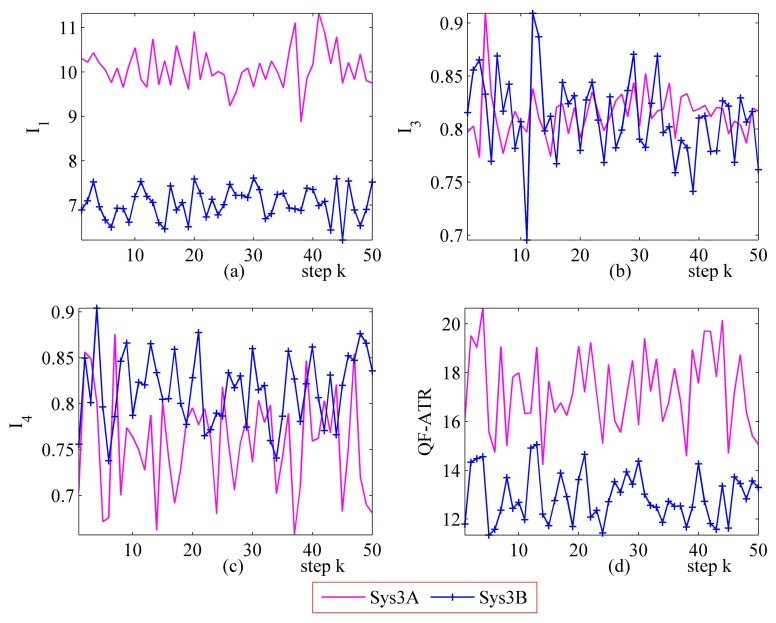
Detailed performance evaluation results of Sys3A and Sys3B for 50 runs, with INR_Sys3A = 0.36, and INR_Sys3B = 0.36. (**a**) Detailed results of *I*_1_; (**b**) Detailed results of *I*_3_; (**c**) Detailed results of *I*_4_; (**d**) Detailed results of *Q*.

**Figure 17.**
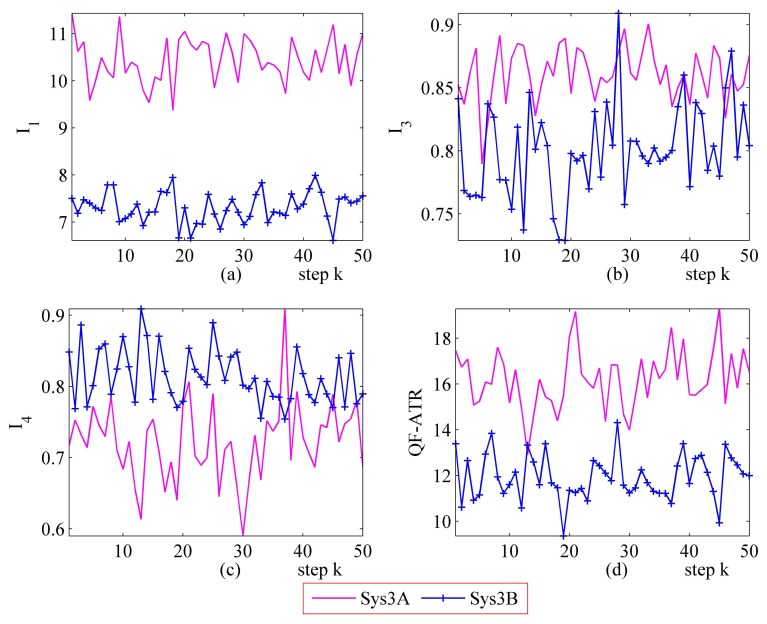
Detailed performance evaluation results of Sys3A and Sys3B for 50 runs, with INR_Sys3A=0.40, and INR_Sys3B=0.40. (**a**) Detailed results of *I*_1_; (**b**) Detailed results of *I*_3_; (**c**) Detailed results of *I*_4_; (**d**) Detailed results of *Q*.

Figures 16 and 17 represent the performance evaluation results on Sys3A and Sys3B. Because the target being recognized is the same one and the designation of these two systems is similar, the actual outputs from Sys3A and Sys3B maintain a similar tendency. However, the performance marks from the proposed method are different. This result confirmed that, even in a challenging evaluation work, the newly developed methodology is suitable for evaluating the ATR system's performance.

In the upper-left part (Figures 16 and 17), the *I*_1_ of Sys3A is much more better than that of Sys3B. For the *I_3_* and the *I*_4_, Sys3A and Sys3B are similar in these two scenarios. In the lower-right part (Figures 16 and 17), it is clear that the QF-ATR of Sys3A overwhelms those of Sys3B.

#### 3.3.4. Further Performance Prediction Results on Sys3A and Sys3B

**Figure 18.**
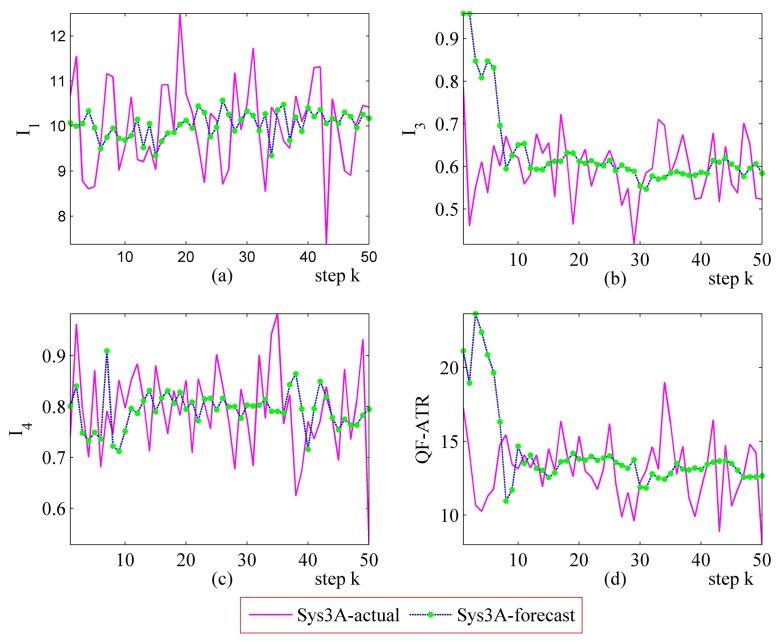
Detailed performance prediction results of Sys3A for 50 runs (INR = 0.36, the number of prediction seeds is 12). (**a**) Predicted and actual *I*_1_; (**b**) Predicted and actual *I*_3_; (**c**) Predicted and actual *I*_4_; (**d**) Predicted and actual *Q*.

**Figure 19.**
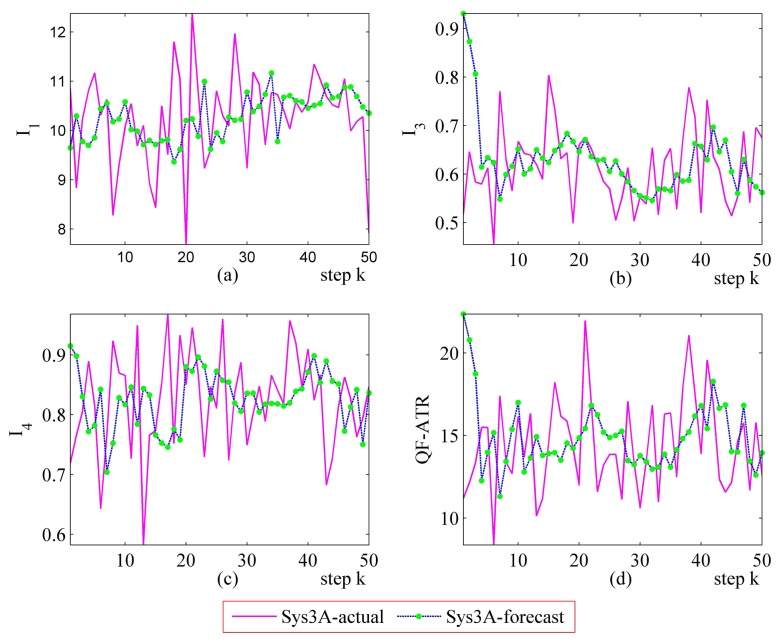
Detailed performance prediction results of Sys3A for 50 runs (INR = 0.36, the number of prediction seeds is 6). (**a**) Predicted and actual *I*_1_; (**b**) Predicted and actual *I*_3_; (**c**) Predicted and actual *I*_4_; (**d**) Predicted and actual *Q*.

**Figure 20.**
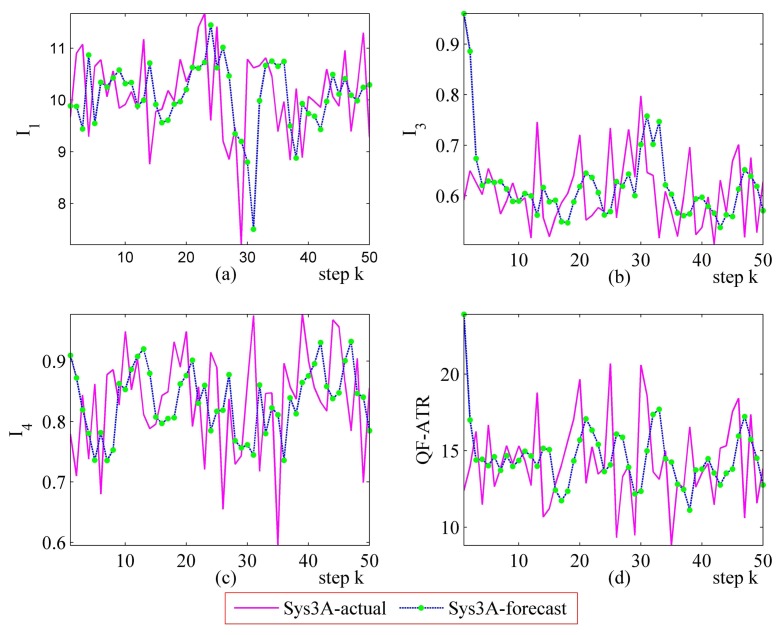
Detailed performance prediction results of Sys3A for 50 runs (INR = 0.36, the number of prediction seeds is 3). (**a**) Predicted and actual *I*_1_; (**b**) Predicted and actual *I*_3_; (**c**) Predicted and actual *I*_4_; (**d**) Predicted and actual *Q*.

**Figure 21.**
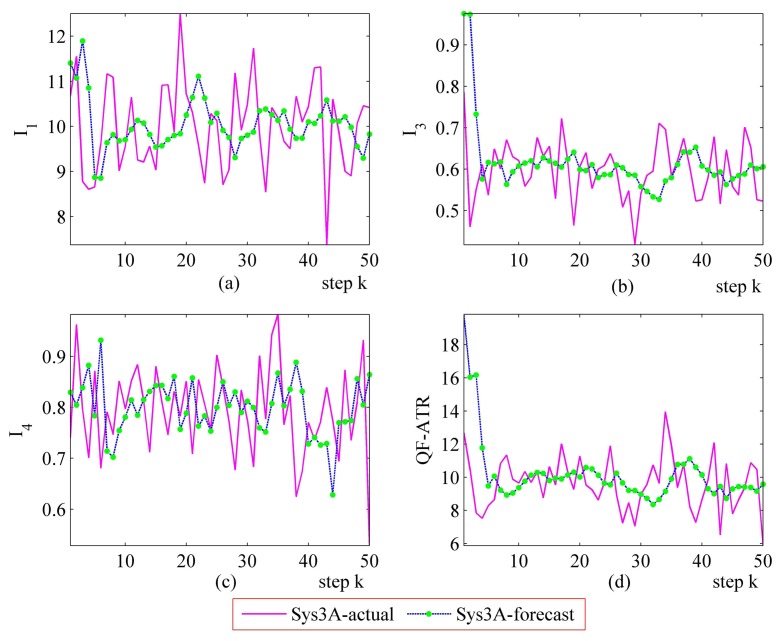
Detailed performance prediction results of Sys3A for 50 runs (INR = 0.49, the number of prediction seeds is 6). (**a**) Predicted and actual *I*_1_; (**b**) Predicted and actual *I*_3_; (**c**) Predicted and actual *I*_4_; (**d**) Predicted and actual *Q*.

**Figure 22.**
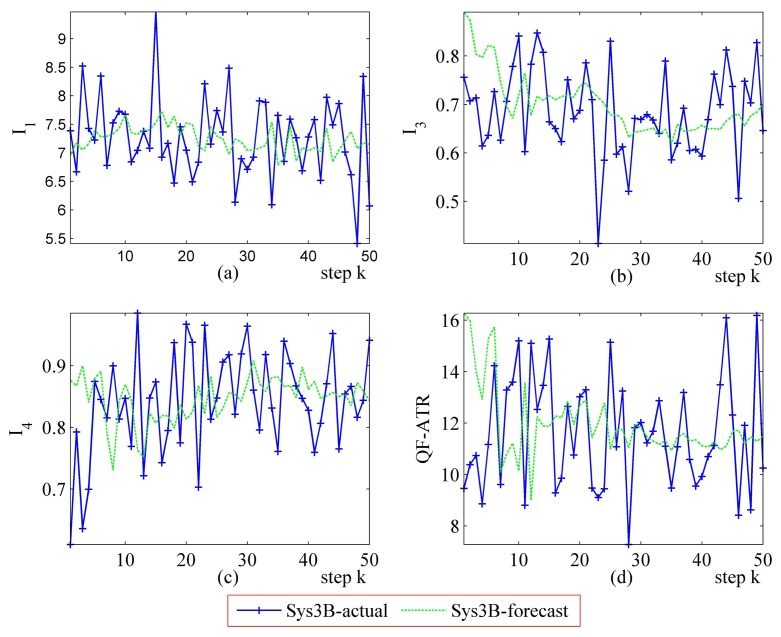
Detailed performance prediction results of Sys3B for 50 runs (INR = 0.36, the number of prediction seeds is 12). (**a**) Predicted and actual *I*_1_; (**b**) Predicted and actual *I*_3_; (**c**) Predicted and actual *I*_4_; (**d**) Predicted and actual *Q*.

**Figure 23.**
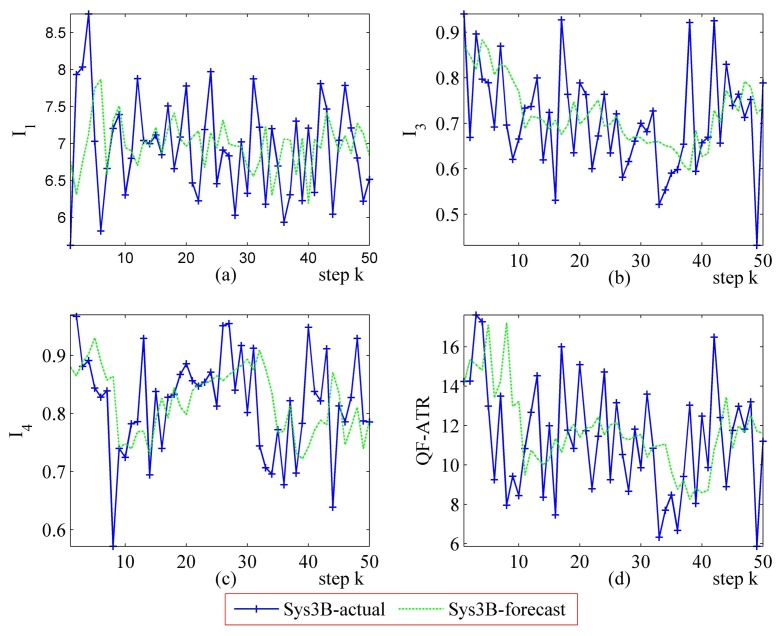
Detailed performance prediction results of Sys3B for 50 runs (INR = 0.36, the number of prediction seeds is 6). (**a**) Predicted and actual *I*_1_; (**b**) Predicted and actual *I*_3_; (**c**) Predicted and actual *I*_4_; (**d**) Predicted and actual *Q*.

**Figure 24.**
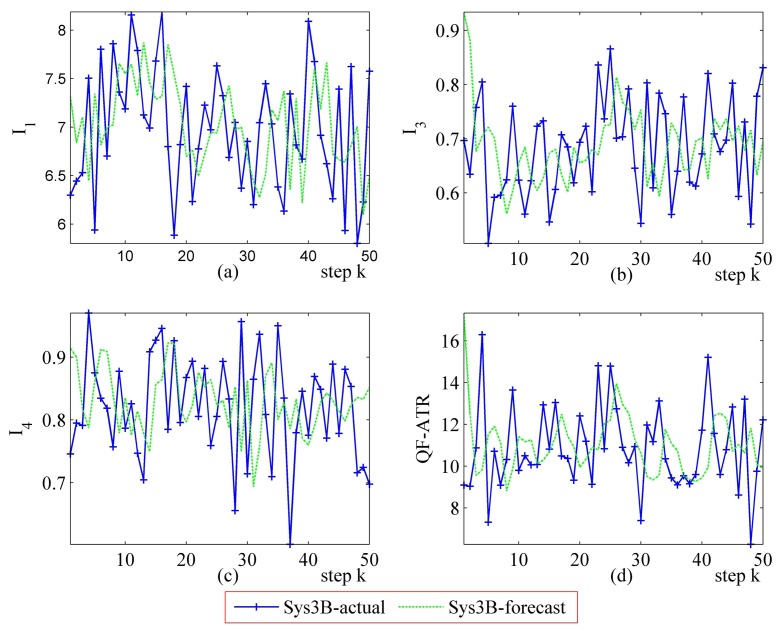
Detailed performance prediction results of Sys3B for 50 runs (INR = 0.36, the number of prediction seeds is 3). (**a**) Predicted and actual *I*_1_; (**b**) Predicted and actual *I*_3_; (**c**) Predicted and actual *I*_4_; (**d**) Predicted and actual *Q*.

**Figure 25.**
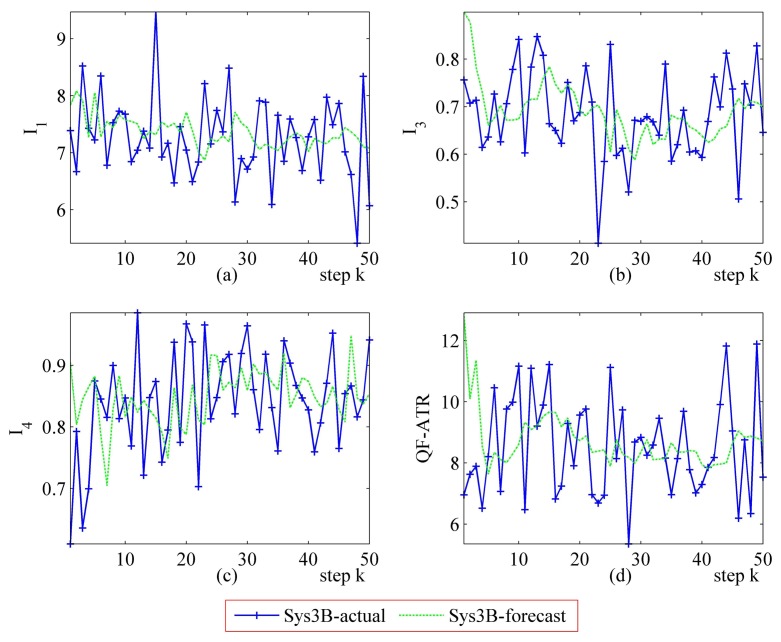
Detailed performance prediction results of Sys3B for 50 runs (INR = 0.49, the number of prediction seeds is 6). (**a**) Predicted and actual *I*_1_; (**b**) Predicted and actual *I*_3_; (**c**) Predicted and actual *I*_4_; (**d**) Predicted and actual *Q*.

Detailed performance prediction results of the above-mentioned Sys3A and Sys3B are given respectively (Figures 18, 19, 20, 21, 22, 23, 24 and 25). These results confirmed that the proposed performance prediction method works well in forecasting the performance of Sys3A and Sys3B. While error exists in individual parts, the predicting accuracy is almost as well as that can be expected.

### 3.4. Comparison between the Existing Technologies and the Proposed Methodologies in Performance Evaluation For an ATR System

Based on the materials presented above, a comparison between the existing technologies and the proposed methodologies is performed in Table 9. As afore-mentioned, most of the existing performance prediction methods are extending work of performance evaluation technologies. Therefore, the comparison between performance prediction methods is not presented. Readers are encouraged to finish this work. The meaning of some symbols are list below.

- **LI**: Is the operating condition considered in the evaluating course?
- **L2**: The objectiveness of the evaluation result.
- **L3**: The effectiveness of the method in revealing the performance from various aspect.
- **L4**: The generalization of the method.
- **L5**: Is the method easy to configure?

### 3.5. Discussion

As can be seen from the aforementioned data, the proposed methodology can offer reasonable performance evaluation and performance prediction results for the ATR systems. To ensure a practical and reliable mechanism, there are still some extended topics related to this work.

First, for some ATR systems, it may be difficult to determine INR. The features for recognition may be indistinct, or cannot be directly converted into variables, e.g., image, voice, smell and similar items which are used to recognize animals cannot be scaled into feature vectors. For signals that cannot be denoted with feature vectors, the INR is set to 1 temporarily for all *s* possible situations; then the system makes use of those signals, and the *s* “faked (because the INR has not been considered)” QF-ATR are arrived at as *Q̃_i_, i* = 1, 2, …, *s*, consequently, for the *i^th^* situation,

(12) $$d(i)=\overset{s}{\underset{i=1}{\text{∑}}}{\tilde{Q}}_{i}/\left({\tilde{Q}}_{i}\overset{s}{\underset{k=1}{\text{∑}}}\overset{s}{\underset{j=1}{\text{∑}}}\frac{{\tilde{Q}}_{j}}{{\tilde{Q}}_{k}}\right)$$

the QF-ATR with INR is then arrived at.

Second, it is meaningful to settle the sample size and know the degree of confidence in a field test. When the risk is assigned in a field test, the sample size and the degree of confidence can be solved by hypothesis testing.

In addition, if the sample size is less than the demand, bootstrapping can offer some help [68,73].

## 4. Conclusions

To sum up, this work offers a comprehensive performance analysis tool for ATR systems. For various system processing an identical target under various condition, the evaluation results by this novel facility can reveal the accomplishment of the system by the evaluation indexes and QF-ATR, as is confirmed by the experimental results. At the same time, it has no limitations and presumptions imposed on the system being considered.

For a given ATR system, the INR index can scale the operating condition in an objective way; the evaluation indexes and the evaluation function serve to interpret the system's accomplishments. The QF-ATR factor, like the quality factor in circuits, may reveal the general capabilities of the entire system. All the proposed methodologies is suitable for all existing ATR systems. However, the methodologies are especially helpful for ATR in radars and photo-sensors.

While convenient to exercise, this methodology is unfamiliar at first sight since it is newly proposed. Although it is still too early to determine whether or not this is the most suitable way to conduct PE-ATR, the results it provides will place PE-ATR on a more objective and quantitative footing. It can also serve as a reference for performance analysis of similar systems.

The future research on this topic may origin from:

- Validation of the methodology with large scale field tests.
- Application in different ATR systems.
- Performance evaluation and performance prediction with less samples.

## Figures and Tables

**Table 1.** For an automatic target recognition system, all the decision types and their names.

**[Info.] in the Feeding Signal**	**The Final Decision of an ATR System**			
	**Target A**	**Other Trained Target**	**Untrained Target**	**No Target**
**Target A**	True [R]	False [R]	False [R]	Omitted [R]
	(*n*_11_)	type I (*n_12_*)	type II (*n*_13_)	(*n*_14_)
**An Untrained Target**	False [D]	False [D]	True [D]	Omitted [D]
	type I (*n*_21_)	type I (*n*_22_)	(*n*_23_)	(*n*_24_)
**No Target**	False [I]	False [I]	False [I]	True [I]
	type I (*n*_31_)	type I (*n*_32_)	type II (*n*_33_)	(*n*_34_)

**Table 2.** Summary of the proposed methodologies.

**Technology**	**The Proposed Methodologies**
SCR	INR. INR is employed to scale the condition for recognition. It is built based on the knowledge of pattern recognition.
PE-ATR	GARO. There are two types of GARO: n-GARO and c-GARO. GARO shows the capability on correct decisions of the ATR system. The related knowledge are statistics and engineering mathematics.
	RRO. RRO reveals how close the distribution of the operating output is likely to the same distribution as that in the training course. RRO is developed based on statistics.
	IRO. IRO estimates the independence of the recognition output to the operating condition. IRO is investigated based on statistics.
	QF-ATR. QF-ATR estimates the comprehensive performance of an ATR system. It is empirically proposed according to the background of ATR and is resolved based on the proposed evaluation indexes.
PP-ATR	CP CP forecasts the performance of an ATR system. CP is developed based on the knowledge of random processes and regression.

**Table 3.** Partial performance evaluation results for the mentioned ATR algorithms.

**Metrics**	**ATR System**			

	**Sys1**	**Sys2**	**Sys3A**	**Sys3B**
**INR** (*d*(*i*_1_))	0.31	0.79	0.36	0.36
**PE index** (*I*_1_/*I*_3_/*I*_4_)	6.14/0.82/0.83	11.03/0.67/0.75	10.06/0.81/0.66	7.02/0.75/0.78
**QF-ATR** (*Q*)	13.99	6.95	14.85	11.33
**RR**	0.71	0.82	0.85	0.83
**PM-FI**	0.76	0.78	0.82	0.82

**Table 4.** Performance forecasting results and validation.

**INR, PE Indexes and QF-ATR**	**ATR System**			

	**Sys1**	**Sys2**	**Sys3A**	**Sys3B**
**INR** (*d*(*i*_1_))	0.31	0.79	0.36	0.36

**PE index** (*I*_1_/*I*_3_/*I*_4_, ▷)	6.16/0.68/0.84	11.04/0.70/0.79	9.92/0.61/0.80	7.16/0.73/0.83
**PE index** (*I*_1_/*I*_3_/*I*_4_, ◁)	6.14/0.65/0.80	10.98/0.69/0.83	9.88/0.61/0.82	7.11/0.73/0.82

**QF-ATR** (*Q*, ▷)	11.24	7.76	14.17	12.11
**QF-ATR** (*Q*, ◁)	10.35	7.97	13.82	11.96

**UB-RR** (▷)	0.75	0.85	0.90	0.90
**LB-RR** (◁)	0.70	0.80	0.85	0.85
**RR**(▷)	0.70	0.83	0.86	0.87

Legend: ▷: forecasted output. ◁: tested result. UB-RR: upper bound of recognition rate. LB-RR: lower bound of recognition rate.

**Table 5.** Performance evaluation results with “Confusion Matrix Method” for the mentioned ATR algorithms Sys1 and Sys2.

**Sys1**					**Sys2**				
	
**Target**	**Omitted [R]**	**Tl**	**T2**	**T3**	**Target**	**Omitted [R]**	**T6**	**T7**	**T9**
Tl	2	443	50	5	T6	0	35	2	1
T2	3	20	475	2	T7	0	1	33	4	
T3	13	6	11	470	T9	1	0	1	36	

**Table 6.** Performance evaluation results with “Confusion Matrix Method” for the mentioned ATR algorithms Sys3A and Sys3B.

**Signal**	**Sys3A**			**Sys3B**		
	
	**Omitted [R]**	**P Pulse**	**T Pulse**	**Omitted [R]**	**P Pulse**	**T Pulse**
P Pulse	2	59	4	5	58	2
T Pulse	3	2	60	0	4	61

**Table 7.** The primary setting of the performance evaluation experiments.

**Metric**	**ATR System**			

	**Sys1**	**Sys2 Sys3A**	**Sys3B**
**INR**	Figure 6, 0.31	Figure 6, 0.79	Figure 16, 0.36
	Figure 7, 0.77	Figure 17, 0.40	Figure 17, 0.40

**Table 8.** The primary setting of the performance prediction experiments.

**Name of the Parameter**	**ATR System**			

	**Sys1**	**Sys2**	**Sys3A**	**Sys3B**
**INR**	0.31	0.79	0.36	0.36

**Number of Prediction Seeds**	Figure 8, 12/Figure 9, 6/Figure 10, 3	Figure 12, 12/Figure 13, 6/Figure 14, 3	Figure 18, 12/Figure 19, 6/Figure 20, 3	Figure 22, 12/Figure 23, 6/Figure 24, 3

**INR**	0.55	0.63	0.49	0.49

**Number of Prediction Seeds**	Figure **11,** 3	Figure 15, 3	Figure 21, 3	Figure 25, 3

**Table 9.** The comparison between the existing technologies and the proposed methodologies in performance evaluation for an ATR system.

**Aspect**	**Existing Technologies**				**Newly Proposed Methodologies**			
	
	**ROC**	**CM**	**RR**	**PM-FI**	**GARO**	**RRO**	**IRO**	**QF-ATR**	
**LI**	**×**	**×**	**×**	✓	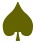	✓	✓	✓
**L2**	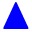	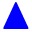	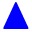	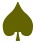	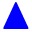	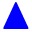	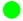	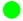
**L3**	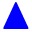	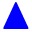	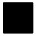	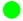	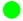	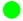	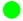	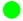
**L4**	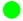	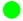	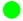	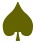	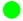	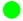	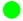	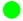
**L5**	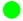	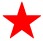	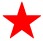	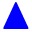	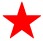	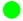	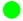	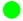

Legend: **The following symbols are effective in all the tables in this work.** 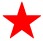: high achievement; 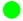: satisfactory; 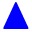: improvement needed; 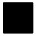 unsatisfactory; 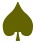: should be considered according to the corresponding method. ✓: yes; ×: no.

## Glossary of Abbreviations

AAF: amplitude affection factor
AR: autoregression
Auto-I: automated instrumentation and evaluation
CFAR: constant false alarm rate
CM: confusion matrix
ECG: electrocardiograph
EEG: electroencephalograph
EMD: empirical mode decomposition
EOC: extended operating condition
EP: expert prediction
ER: extend of recognition
FPGA: flexible field programmable gate array
IR: infrared
LB-RR: lower bound of recognition rate
MFRR: measurement of false recognition rate
MOE: measures of effectiveness
MRR: measurement of recognition rate
NN: neural network
PE: performance evaluation
PE-ATR: performance evaluation for ATR systems
PM-FI: performance models based on fuzzy integration
POLInSAR: polarimetric SAR interferometry
PP-ATR: performance prediction for ATR systems
QDD: quadratic distance discriminator
ROC: receiver operating characteristic
RR: recognition rate
SAR: synthetic aperture radar
SCR: scaling the condition for recognition
SNR: signal to noise ratio
SNRF: signal to noise ratio affect factor
START: the scoring, truthing, and registration toolkit
UB-RR: upper bound of recognition rate
UCI: University of California Irvine
WQDD: weighted quadratic distance discriminator
